# Preclinical Evidence of Berberine on Non-Alcoholic Fatty Liver Disease: A Systematic Review and Meta-Analysis of Animal Studies

**DOI:** 10.3389/fphar.2021.742465

**Published:** 2021-09-09

**Authors:** Sichen Ren, Xiao Ma, Ruilin Wang, Honghong Liu, Ying Wei, Shizhang Wei, Manyi Jing, Yanling Zhao

**Affiliations:** ^1^ Department of Pharmacy, The Fifth Medical Center of Chinese PLA General Hospital, Beijing, China; ^2^ School of Pharmacy, Chengdu University of Traditional Chinese Medicine, Chengdu, China; ^3^ Integrative Medical Center, The Fifth Medical Center of Chinese PLA General Hospital, Beijing, China

**Keywords:** berberine, NAFLD/NASH, meta-analysis, preclinical evidence, animal studies

## Abstract

As lifestyle and diet structure impact our health, non-alcoholic fatty liver disease (NAFLD) is prevalent all over the world. Some phytomedicines containing berberine (BBR) have been extensively used for centuries in Ayurvedic and traditional Chinese medicine. The goal of this systematic review is to investigate the preclinical evidence of BBR on NAFLD models. The following relevant databases, including Web of Science, PubMed, the Cochrane Library, and Embase, were retrieved from inception to May 2021. The content involved BBR on different animal models for the treatment of NAFLD. The SYstematic Review Center for Laboratory animal Experimentation (SYRCLE) Animal Experiment Bias Risk Assessment Tool was used to assess the methodological quality and RevMan 5.4 software was used to conduct the meta-analysis based on the Cochrane tool. A total of 31 studies involving 566 animals were included, of which five models and five animal breeds were reported. The results showed that TC, TG, ALT, AST, HDL-C, LDL-C, FBG, FINS, and FFA in the group treated with BBR were significantly restored compared with those in the model group. HOMA-IR had a significant downward trend, but the result was not significantly different (P = 0.08). The subgroup analysis of the different models and different animal breeds indicated that BBR could ameliorate the aforementioned indicator levels, although some results showed no significant difference. Finally, we summarized the molecular mechanisms by which berberine regulated NAFLD/NASH, mainly focusing on activating the AMPK pathway, improving insulin sensitivity and glucose metabolism, regulating mitochondrial function, reducing inflammation and oxidative stress, regulating cell death and ER stress, reducing DNA methylation, and regulating intestinal microenvironment and neurotoxicity. The preclinical evidence suggested that BBR might be an effective and promising drug for treating NAFLD/NASH. In addition, further studies with more well-designed researches are needed to confirm this conclusion.

## Introduction

Obesity with extensive metabolic regulation disorders and excessive fat accumulation is a major risk factor for type 2 diabetes, various kinds of cancer, cardiovascular disease, and non-alcoholic fatty liver disease (NAFLD) (Sun et al., 2017). As the lifestyle and diet structure translate, obesity is prevalent all over the world. As high as 30 percent of the population in western developed countries suffer from NAFLD (Williams, 2006; Angulo, 2007) and the prevalence is on the rise in developing countries (close to 10%) (Fan et al., 2009). As the aggressive form of NAFLD, non-alcoholic steatohepatitis (NASH) can further develop into hepatic cirrhosis and even hepatocellular cancer without appropriate treatment and is increasingly recognized as the major reason for liver transplantation or end-stage liver disease (Zhang et al., 2016; Goldberg et al., 2017). At present, the pathogenesis of NAFLD/NASH is still not clear enough, but the “two-hit theory” and “multi-parallel hits theory” are widely accepted explanations (Day et al., 1998; Malaguarnera et al., 2009). Excessive hepatic fat accumulation, oxidative stress, insulin resistance or others play promoting roles in developing NAFLD/NASH (Day et al., 1998; Tilg et al., 2010). Therefore, treatment based on these theories may be extremely important in preventing NASH.

Due to the serious impact on the quality of human life and longevity and the high incidence, NAFLD has been receiving more and more attention worldwide. In fact, lifestyle change with exercise and diet intervention to ameliorate insulin sensitivity is the best treatment for NAFLD but limited by compliance and persistence difficulties, emphasizing the urgent need for pharmacotherapy (Scherer et al., 2016; Sumida et al., 2018). Unfortunately, no medication available for NAFLD/NASH is approved. At present, pioglitazone, metformin, vitamin E, and other pharmacotherapies are usually taken in the clinic to treat NAFLD, but they often had limited effects and critical side effects (Wong et al., 2014; Sumida et al., 2018).

**FIGURE 1:**
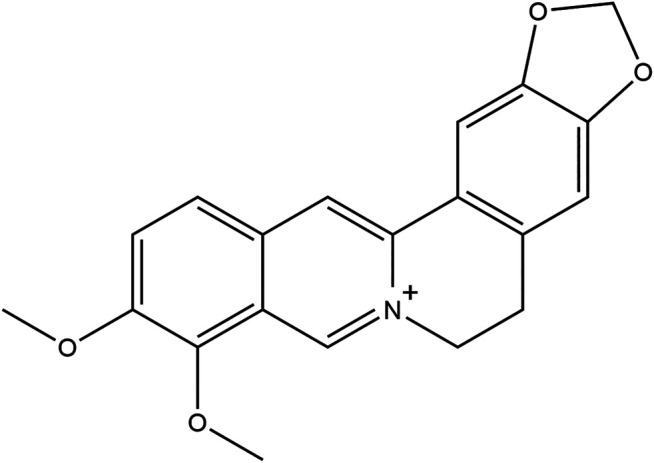
The chemical structure of berberine.

Berberine (BBR, Figure 1), a protoberberine isoquinoline alkaloid isolated from many species of plants, such as *Berberis aquifolium* or *Coptis chinensis*, which has been extensively used for gut infections and diarrhea for centuries as a part of Ayurvedic and traditional Chinese medicine (Tillhon et al., 2012; Zhu et al., 2019). Over the past decades, it has been reported that BBR has various pharmacological effects of lowering blood glucose, lowering blood lipid, and improving insulin sensitivity and glucose tolerance, and anti-inflammatory, anti-oxidant, and anti-diabetic effects (Kong et al., 2004; Lee et al., 2006; Wang et al., 2011; Zhao et al., 2012; Mo et al., 2014; Sun et al., 2018).

At present, accumulated evidence from the clinical efficacy and pharmacological effect indicated that BBR may have great potential in treating NAFLD. Nevertheless, there are different types of NAFLD/NASH with various manifestations. In order to compensate for the disparity between the clinical efficacy and pharmacological effect or study BBR on a wide range of NAFLD/NASH and its further mechanism, it is necessary to investigate the preclinical evidence of BBR on various NAFLD/NASH.

## Methods

### Literature Search and Review Strategy

We searched relevant databases, including Web of Science, PubMed, the Cochrane Library, and Embase, from inception to May 2021. The main search terms were combinations of “berberine” and “Non alcoholic fatty liver disease” or “Non-alcoholic fatty liver disease” or “Nonalcoholic fatty liver disease” or “NAFLD” or “non alcoholic steatohepatitis” or “non-alcoholic steatohepatitis” or “nonalcoholic steatohepatitis” or “NASH” or “metabolic associated fatty liver disease” or “MAFLD” in various databases. Moreover, the main goal is to gain documents that match most of the keywords. A preliminary screening of the retrieved literature was performed using Endnote software (Version X9.1, Thompson Reuters) to remove duplicate records. Subsequently, two researchers (Sichen Ren and Ying Wei) independently reviewed the title/abstract related to the topic. A full-text read of potential documents that met the eligibility criteria was conducted. Any disagreements between the two researchers were resolved through negotiation or third-party consensus.

### Inclusion and Exclusion Criteria

Studies were included if they met all of the following criteria: 1) the experiment was based on the NAFLD/NASH model only; 2) only the BBR experimental group was included and it received BBR only; 3) the included studies consist of a model group and BBR experimental group; 4) the primary endpoints were as follows: total cholesterol (TC), triglycerides (TG), alanine aminotransferase (ALT), aspartate aminotransferase (AST), high-density lipoprotein cholesterol (HDL-C), low-density lipoprotein cholesterol (LDL-C), fasting blood glucose (FBG), fasting insulin (FINS), homeostasis model assessment-insulin resistance (HOMA-IR), and free fatty acid (FFA) and the secondary endpoints were as follows: animal body weight, liver weight, liver index, NAFLD activity score (NAS), steatosis score, tumor necrosis factor-α (TNF-α), interleukin-6 (IL-6), interleukin-1β (IL-1β), glutathione (GSH), and thiobarbituric acid reactive substances (TBARS); 4) the language is limited to English.

The exclusion criteria of this study were as follows: 1) irrelevant or duplicate publications; 2) no animal experiment included; 3) experimental group without BBR and NAFLD/NASH or other measures out of BBR and NAFLD/NASH; 4) review and/or meta-analysis; 5) insufficient primary and secondary outcome data; 6) RCT or clinical research; 6) book, thesis, or conference proceedings. Thus, the investigators screened the articles initially by title or abstract based on the inclusion criteria. For example, the relevant literature on BBR with curcumin for the treatment of NAFLD was not included.

### Data Extraction

We independently assessed all the included studies and extracted the following data using a standardized data extraction form: 1) the name of the first author and the year of publication; 2) numbers of animals in the model group and BBR experimental group; 3) the model of NAFLD/NASH and BBR dosage; 4) the primary and second outcome measures. If the experimental group of animals in a certain study were measured several times at different time points after BBR administration, all data were extracted and the dominant dose of BBR was included. If details of the data are insufficient, the publishers were contacted for further information. Information of all studies is shown in Supplementary Table S1.

### Assessment of Quality

The SYstematic Review Center for Laboratory animal Experimentation (SYRCLE) Animal Experiment Bias Risk Assessment Tool was used for assessing the methodological quality. The tool comprises 10 sections related to selection bias, performance bias, detection bias, attrition bias, reporting bias, and other biases. Moreover, the assessment score of each section was yes (low risk of bias), no (high risk of bias), and unclear (the risk of bias is insufficient to assess from the reported details). These ten terms are as follows: 1) the allocation sequence was fully generated or applied; 2) the groups were similar at baseline; 3) the allocation was fully concealed; 4) the animals were randomly placed during the experiment; 5) the investigators are blinded during the experiment; 6) the animals were selected at random for results; 7) the result assessor was blinded; 8) incomplete data were fully addressed; 9) the studies were free of selective results reporting; (10) the study was free of other problems that could result in high risk of bias (Peters et al., 2006; Hooijmans et al., 2014). Any disagreements between the two researchers during the assessment were resolved through negotiation or third-party consensus.

### Statistical Statistics

All data were analyzed using the Review Manager (RevMan, version 5.4). Before determining the pooled effect, I-square (I^2^) test and *Q*-test were used to assess heterogeneity between studies. Based on the results of the Q-test and I^2^ test, a fixed-effects model with minor heterogeneity (I^2^ ≤ 50% or *p* ≥ 0.1) or a random-effects model with significant heterogeneity (I^2^ > 50% or *p* < 0.1) were chosen. All outcomes were presented as the std. mean difference (SMD) with a 95% confidence interval (*CI*) and the significance of pooled effects was determined by the *Z*-test. *p* < 0.05 was considered statistically significant.

## Results

### Study Identification and Selection

**FIGURE 2:**
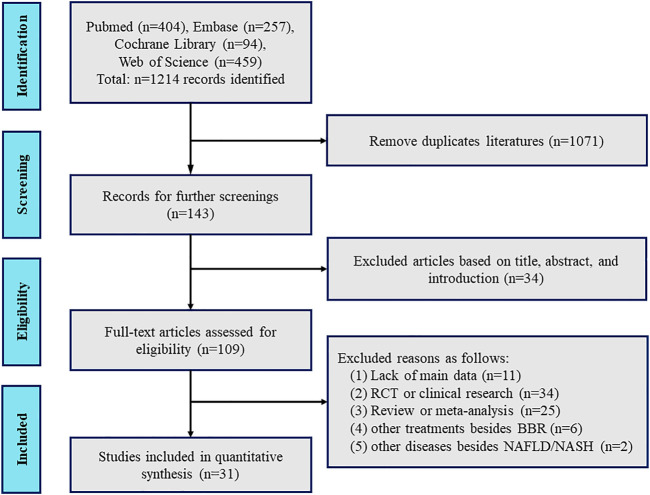
Flowchart of the study selection process.

As shown in Figure 2, a flow diagram of the system evaluation was constructed. A total of 1,214 relevant studies were identified based on our search strategy. After excluding duplicate literature (1,071 records) and irrelevant documents (34 records), 109 articles were left for further full-text assessment. Ultimately, 31 studies were included for further quality assessment and meta-analysis according to our exclusion criteria.

### Study Characteristics

In total, 566 animals (285 in the model group; 281 in the BBR group) in 31 studies were included in this meta-analysis. Moreover, Sprague Dawley (SD) rats, Wistar rats, albino rats, C57BL/6 mice, or C57BLKS/J-*Lepr* ^db^/*Lepr* ^db^ (db/db) mice were reported in these included studies. The animal models included feeding a high-fat diet (HFD), or methionine- and choline-deficient (MCD) diet, or db/db mice. Interestingly, there were also intraperitoneal injections of tunicamycin (TM) and CCl_4_ models. The BBR group were mainly treated intragastrically with BBR at dosages ranging from 50 mg/kg to 300 mg/kg, while two studies have reported that animals were intraperitoneally injected with 5 mg/kg BBR. Meanwhile, one study has reported adding 1.4 g/kg BBR to animals’ HFD. The duration of administration ranged from 3 days to 20 weeks. The main reason for this was due to the different modeling methods and BBR dosage. Moreover, the outcomes included main outcome measures such as TC, TG, ALT, AST, HDL-C, LDL-C, FFA, FBG, FINS, and HOMA-IR and other outcome measures such as animal body weight, liver weight, liver index, NAS, steatosis score, TNF-α, IL-6, IL-1β, GSH, and TBARS. Few adverse reactions of animals were reported in the included studies; see Supplementary Table S1 for details.

### Methodological Quality

**FIGURE 3:**
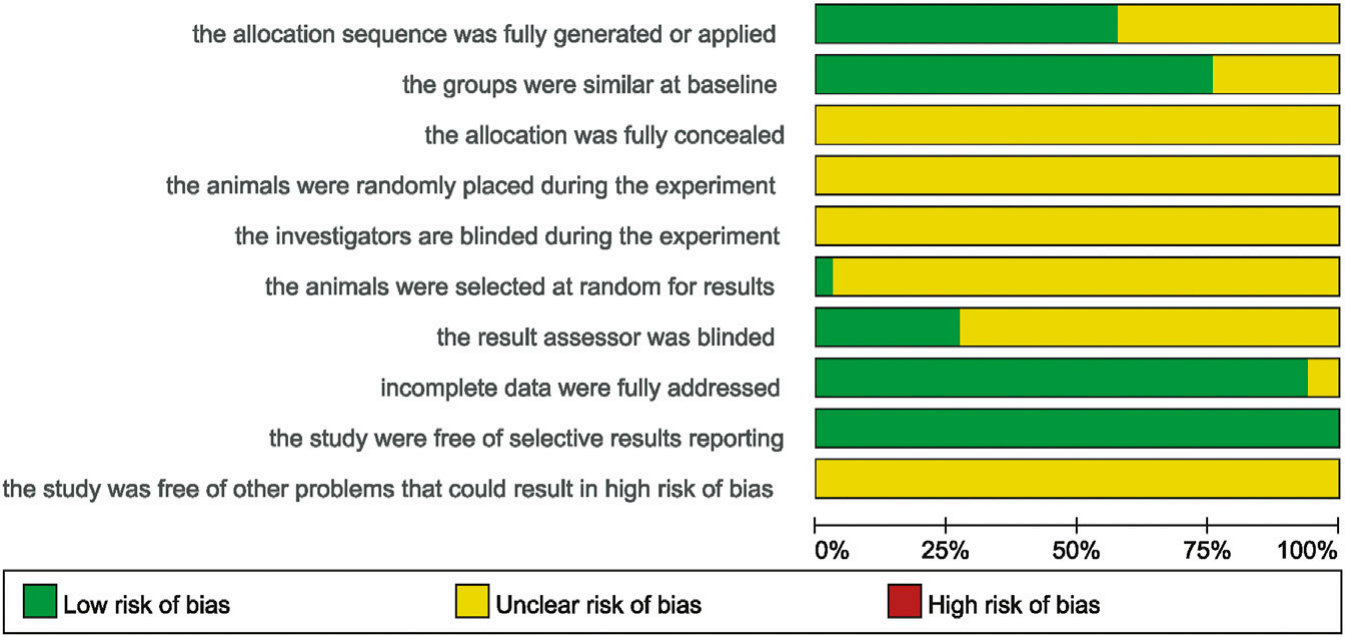
The risk of bias graph of included studies.

Nineteen studies were fully generated or applied the randomized allocation sequence. Twenty-five studies have reported that the groups were similar at baseline. Only one study described the animals that were selected at random for results. Seven studies described blinded result assessment. Only two studies described incomplete data and all studies were free of selective results reporting. However, the following aspects were not clear: fully concealed allocation, random housing of the animals, blinded investigators, and being free of other bias. All the details are shown in Figure 3 and Supplementary Figure S1.

## Effect of BBR Treatment on NAFLD

### Major Outcome Measures

#### Lipids Content Analysis

23 studies with 426 animals reported the blood TC level after BBR treatment for NAFLD/NASH. Blood TC showed significant heterogeneity (*p* < 0.00001, *I* ^*2*^ = 85%). The random-effects model was used in the meta-analysis. BBR could observably reduce the blood TC level compared with the model group [*SMD* = 2.23, 95% *CI* (1.56, 2.90), *p* < 0.00001] (Table 1; Supplementary Figure S2A). Eight studies with 133 animals have reported the liver tissue TC level after BBR treatment for NAFLD/NASH. Liver tissue TC was found to have minor heterogeneity (*p* = 0.17, *I* ^*2*^ = 32%). Therefore, we used the fixed-effects model in the meta-analysis. BBR could remarkably decrease the liver tissue TC levels compared with the model group [*SMD* = 1.70, 95% *CI* (1.27, 2.12), *p* < 0.00001] (Table 1; Supplementary Figure S2B).

**TABLE 1:** Comparison of major outcome measures of RUT treatment for NAFLD/NASH.

Measures	Studies	Model group	BBR group	Heterogeneity		*SMD* (95%CI)	*p*-value
				*p*	*I*^*2*^ (%)		
Blood TC	23	210	216	<0.00001	85	2.23 (1.56, 2.90)	<0.00001
Liver TC	8	65	68	0.17	32	1.70 (1.27, 2.12)	<0.00001
Blood TG	24	210	216	<0.00001	88	2.58 (1.78, 3.37)	<0.00001
Liver TG	14	114	117	0.01	53	1.99 (1.49, 2.49)	<0.00001
HDL-C	12	108	111	<0.00001	83	−1.04 (−1.82, −0.26)	0.009
LDL-C	15	146	149	<0.00001	86	2.56 (1.69, 3.43)	<0.00001
FFA	5	45	37	<0.00001	89	2.66 (0.44, 4.88)	0.02
ALT	22	197	192	<0.00001	86	2.63 (1.86, 3.41)	<0.00001
AST	21	190	185	<0.00001	86	2.36 (1.61, 3.12)	<0.00001
FBG	11	88	92	<0.00001	86	1.77 (0.74, 2.81)	0.0008
FIN	8	70	71	<0.0001	78	2.23 (1.24, 3.23)	<0.0001
HOMA-IR	4	36	37	<0.00001	92	2.75 (−0.35, 5.85)	0.08

24 studies with 426 animals have reported the blood TG level after BBR treatment for NAFLD/NASH. There was obvious heterogeneity in blood TG level (*p* < 0.00001, *I* ^*2*^ = 88%). So, the random-effects model was chosen in the meta-analysis. Compared with the model group, the blood TG level was clearly different [*SMD* = 2.58, 95% *CI* (1.78, 3.37, *p* < 0.00001] (Table 1; Supplementary Figure S3A). 14 studies with 231 animals reported the liver tissue TG level after BBR treatment. Liver tissue TG was analyzed to have significant heterogeneity (*p* = 0.01, *I* ^*2*^ = 53%). The random-effects model was used in the meta-analysis. Compared with the model group, BBR could remarkably reduce the liver tissue TG level [*SMD* = 1.99, 95% *CI* (1.49, 2.49), *p* < 0.00001] (Table 1; Supplementary Figure S3B).

12 studies with 219 animals have reported the HDL-C level after BBR treatment for NAFLD/NASH. There was outstanding heterogeneity in HDL-C level (*p* < 0.00001, *I* ^*2*^ = 83%). Thus, we used the random-effects model in the meta-analysis. BBR could observably increase the HDL-C level compared with the model group [*SMD* = −1.04, 95% *CI* (−1.82, −0.26), *p* = 0.0009] (Table 1; Supplementary Figure S4A). 15 studies with 275 animals have reported the LDL-C level after BBR treatment for NAFLD/NASH. There was significant heterogeneity in LDL-C level (*p* < 0.00001, *I* ^*2*^ = 86%). The random-effects model was utilized for the meta-analysis. Compared with the model group, the LDL-C level was significantly different [*SMD* = 2.56, 95% *CI* (1.69, 3.43), *p* < 0.00001] (Table 1; Supplementary Figure S4B). Five studies with 82 animals have reported the FFA level after BBR treatment for NAFLD/NASH. FFA level was found remarkably heterogeneous (*p* < 0.00001, *I* ^*2*^ = 89%). Therefore, we took the random-effects model in the meta-analysis. BBR could apparently lessen FFA compared with the model group [*SMD* = 2.66, 95% *CI* (0.44, 4.88), *p* = 0.02] (Table 1; Supplementary Figure S4C). These results have shown that BBR could restore the lipids content and reduce liver injury.

#### Liver Function Analysis

22 studies with 389 animals have reported the ALT level after BBR treatment for NAFLD/NASH. There was remarkable heterogeneity in ALT level (*p* < 0.00001, *I* ^*2*^ = 86%). Hence, we adopted the random-effects model in the meta-analysis. Compared with the model group, BBR caused a sharp decline in the ALT level [*SMD* = 2.63, 95% *CI* (1.86, 3.41), *p* < 0.00001] (Table 1; Supplementary Figure S5A). 21 studies with 373 animals reported the AST level after BBR treatment for NAFLD/NASH. AST level was demonstrated to be significantly heterogeneous (*p* < 0.00001, *I* ^*2*^ = 86%) so as to select the random-effects model. BBR could evoke an apparent reduction in the AST level compared with the control group [*SMD* = 2.36, 95% *CI* (1.61, 3.12), *p* < 0.00001] (Table 1; Supplementary Figure S5B). The above results have indicated that BBR could effectively ameliorate liver function and alleviate liver injury.

#### Insulin Resistance and Glucose Metabolism Analysis

11 studies with 180 animals have reported the FBG level after BBR treatment for NAFLD/NASH. There was obvious heterogeneity in FBG level (*p* < 0.00001, *I* ^*2*^ = 86%), so we took the random-effects model in the meta-analysis. The results have indicated that BBR could significantly alleviate the FBG level compared with the control group [*SMD* = 1.77, 95% *CI* (0.74, 2.81), *p* = 0.0008] (Table 1; Supplementary Figure S6A). Eight studies with 141 animals have reported the FINS level after BBR treatment for NAFLD/NASH. It was memorably heterogeneous in the FINS level (*p* < 0.00001, *I* ^*2*^ = 78%); thus, we adopted the random-effects model for the meta-analysis. As shown in Table 1 and Supplementary Figure S6B, BBR could effectively reduce the level of FINS compared with the model group [*SMD* = 2.23, 95% *CI* (1.24, 3.23), *p* < 0.00001]. Four studies with 56 animals reported the HOMA-IR index after BBR treatment for NAFLD/NASH. Obviously, there was remarkable heterogeneity in the HOMA-IR index (*p* < 0.00001, *I* ^*2*^ = 92%). Moreover, we used the random-effects model in the meta-analysis. However, although we could observe a decreasing trend of HOMA-IR index after BBR treatment, it has no significant difference compared with the model group [*SMD* = 2.75, 95% *CI* (−0.35, 5.85), *p* = 0.08] (Table 1 and Supplementary Figure S6C). The pooled analysis of insulin resistance and glucose metabolism demonstrated that BBR can not only accelerate the recovery of insulin resistance but also improve glucose metabolism and alleviate liver damage.

### Other Outcome Measures

19 studies with 320 animals have reported the animal body weight after BBR treatment for NAFLD/NASH. There was remarkable heterogeneity in body weight (*p* < 0.00001, *I* ^*2*^ = 82%). Hence, we adopted the random-effects model in the meta-analysis. Compared with the model group, BBR caused a sharp decline in the body weight [*SMD* = 1.98, 95% *CI* (1.28, 2.67), *p* < 0.00001] (Supplementary Figure S7A). Eight studies with 122 animals have reported the animal liver weight after BBR treatment for NAFLD/NASH. The animal liver weight was demonstrated to be significantly heterogeneous (*p* = 0.0005, *I* ^*2*^ = 73%) so as to select the random-effects model. BBR could evoke an apparent reduction in the liver weight compared with the control group [*SMD* = 1.17, 95% *CI* (0.35, 2.00), *p* = 0.005] (Supplementary Figure S7B). Five studies with 82 animals reported the liver index after BBR treatment for NAFLD/NASH. Obviously, there was remarkable heterogeneity in the liver index (*p* = 0.0006, *I* ^*2*^ = 79%). Moreover, we used the random-effects model in the meta-analysis. However, although we could observe a decreasing trend of the liver index after BBR treatment, it has no significant difference compared with the model group [*SMD* = 1.12, 95% *CI* (−0.01, 2.25), *p* = 0.05] (Supplementary Figure S7C). Six studies with 111 animals reported the NAS after BBR treatment for NAFLD/NASH. There was obvious heterogeneity in NAS (*p* = 0.0008, *I* ^*2*^ = 76%), so we took the random-effects model in the meta-analysis. The results indicated that BBR could significantly alleviate the NAS compared with the control group [*SMD* = 3.80, 95% *CI* (2.26, 5.23), *p* < 0.00001] (Supplementary Figure S7D). Three studies with 59 animals have reported the steatosis score after BBR treatment for NAFLD/NASH. It was not memorably heterogeneous in the steatosis score (*p* = 0.76, *I* ^*2*^ = 0%); thus, we adopted the fixed-effects model for the meta-analysis. As shown in Supplementary Figure S7E, BBR could effectively reduce the steatosis score compared with the model group [*SMD* = 1.34, 95% *CI* (0.76, 1.92), *p* < 0.00001].

Four studies with 88 animals have reported the TNF-α level after BBR treatment for NAFLD/NASH. There was obvious heterogeneity in TNF-α level (*p* < 0.00001, *I* ^*2*^ = 95%), so we took the random-effects model in the meta-analysis. The results indicated that BBR could significantly alleviate the TNF-α level compared with the control group [*SMD* = 7.00, 95% *CI* (1.58, 12.41), *p* = 0.01] (Supplementary Figure S8A). Three studies with 44 animals have reported the liver TNF-α level after BBR treatment for NAFLD/NASH. It was memorably heterogeneous in the liver TNF-α level (*p* = 0.0002, *I* ^*2*^ = 88%); thus, we adopted the random-effects model for the meta-analysis. As shown in Supplementary Figure S8B, BBR could effectively reduce the level of liver TNF-α compared with the model group [*SMD* = 3.31, 95% *CI* (0.39, 6.22), *p* = 0.03].

Three studies with 68 animals have reported the IL-6 level after BBR treatment for NAFLD/NASH. It was memorably heterogeneous in the IL-6 level (*p* < 0.00001, *I* ^*2*^ = 96%); thus, we adopted the random-effects model for the meta-analysis. As shown in Supplementary Figure S8C, BBR could effectively reduce the level of IL-6 compared with the model group [*SMD* = 7.08, 95% *CI* (1.02, 13.14), *p* = 0.02]. Two studies with 28 animals have reported the IL-1β level after BBR treatment for NAFLD/NASH. It was not heterogeneous in the IL-1β level (*p* = 0.23, *I* ^*2*^ = 30%); thus, we adopted the fixed-effects model for the meta-analysis. As shown in Supplementary Figure S8D, BBR could effectively reduce the level of IL-1β level compared with the model group [*SMD* = 1.83, 95% *CI* (0.89, 2.78), *p* = 0.0001]. Three studies with 68 animals have reported the GSH level after BBR treatment for NAFLD/NASH. Obviously, there was remarkable heterogeneity in the GSH level (*p* = 0.04, *I* ^*2*^ = 77%). Moreover, we used the random-effects model in the meta-analysis. The results indicated that BBR could significantly increase the GSH level compared with the control group [*SMD* = −6.51, 95% *CI* (−10.15, −2.88), *p* = 0.0004] (Supplementary Figure S8E). Three studies with 50 animals have reported the TBARS level after BBR treatment for NAFLD/NASH. Obviously, there was remarkable heterogeneity in the TBARS level (*p* = 0.10, *I* ^*2*^ = 57%). Furthermore, we used the random-effects model in the meta-analysis. The results indicated that BBR could significantly alleviate the TBARS level compared with the control group [*SMD* = 3.13, 95% *CI* (1.74, 4.52), *p* < 0.0001] (Supplementary Figure S8F). The pooled analysis results indicated that BBR can not only accelerate the recovery of inflammation but also alleviate oxidative damage.

### Subgroup Analysis of Related Major Indicators

#### Subgroup Analysis for Different Animal Breeds

13 studies with 236 SD rats have reported the blood TC level after BBR treatment for NAFLD/NASH. There was obvious heterogeneity in blood TC level (*p* < 0.00001, *I* ^*2*^ = 84%), so we took the random-effects model in the meta-analysis. The results indicated that BBR could significantly alleviate the blood TC level compared with the control group [*SMD* = 3.08, 95% *CI* (2.08, 4.08), *p* < 0.00001] (Supplementary Figure S9A 2.1.1). Four studies with 71 C57BL/6 mice have reported the blood TC level after BBR treatment for NAFLD/NASH. It was memorably heterogeneous in the blood TC level (*p* = 0.0002, *I* ^*2*^ = 85%); thus, we adopted the random-effects model for the meta-analysis. However, it was not significantly different compared with the model group [*SMD* = 0.95, 95% *CI* (−0.47, 2.31), *p* = 0.20], as shown in Supplementary Figure S9A 2.1.2. In addition, two studies with 40 Wistar rats have reported the blood TC level after BBR treatment for NAFLD/NASH. Obviously, there was remarkable heterogeneity in the blood TC level (*p* = 0.0006, *I* ^*2*^ = 91%). Moreover, we used the random-effects model in the meta-analysis. However, although we could observe a decreasing trend of blood TC level after BBR treatment, it was not significantly different compared with the model group [*SMD* = 0.56, 95% *CI* (−1.85, 2.97), *p* = 0.65] (Supplementary Figure S9A 2.1.3). Four studies with 68 SD rats have reported the liver tissue TC level after BBR treatment for NAFLD/NASH. There was heterogeneity in liver tissue TC level (*p* = 0.09, *I* ^*2*^ = 54%). Hence, we adopted the random-effects model in the meta-analysis. Compared with the model group, BBR caused a sharp decline in the liver tissue TC level [*SMD* = 1.89, 95% *CI* (0.97, 2.80), *p* < 0.0001] (Supplementary Figure S9B 2.2.1). Two studies with 35 C57BL/6 mice have reported the liver tissue TC level after BBR treatment for NAFLD/NASH. The liver tissue TC level was demonstrated to be significantly heterogeneous (*p* = 0.08, *I* ^*2*^ = 68%) so as to select the random-effects model. BBR could evoke an apparent reduction in the liver tissue TC level compared with the control group [*SMD* = 1.63, 95% *CI* (0.51, 3.11), *p* = 0.03] (Supplementary Figure S9B 2.2.2).

14 studies with 240 SD rats have reported the blood TG level after BBR treatment for NAFLD/NASH. Blood TG showed significant heterogeneity (*p* < 0.00001, *I* ^*2*^ = 92%). The random-effects model was used in the meta-analysis. BBR could observably reduce the blood TG level compared with the model group [*SMD* = 3.64, 95% *CI* (2.21, 5.08), *p* < 0.00001] (Supplementary Figure S10A 2.3.1). Four studies with 67 C57BL/6 mice have reported the blood TG level after BBR treatment for NAFLD/NASH. Blood TG was found to have significant heterogeneity (*p* = 0.03, *I* ^*2*^ = 65%). Therefore, we used the random-effects model in the meta-analysis. BBR could remarkably decrease the blood TG levels compared with the model group [*SMD* = 1.70, 95% *CI* (0.65, 2.74), *p* = 0.001] (Supplementary Figure S10A 2.3.2). Two studies with 40 Wistar rats have reported the blood TG level after BBR treatment for NAFLD/NASH. There was obvious heterogeneity in blood TG level (*p* = 0.005, *I* ^*2*^ = 88%). So, the random-effects model was chosen in the meta-analysis. However, compared with the model group, the blood TG level was not different [*SMD* = 1.16, 95% *CI* (−0.92, 3.24, *p* = 0.27] (Supplementary Figure S10A 2.3.3). Five studies with 84 SD rats have reported the liver tissue TG level after BBR treatment. Liver tissue TG was analyzed to have significant heterogeneity (*p* = 0.03, *I* ^*2*^ = 64%). The random-effects model was used in the meta-analysis. Compared with the model group, BBR could remarkably reduce the liver tissue TG level [*SMD* = 1.88, 95% *CI* (0.96, 2.80), *p* < 0.0001] (Supplementary Figure S10B 2.4.1). Five studies with 83 C57BL/6 mice have reported the liver tissue TG level after BBR treatment. Liver tissue TG was analyzed to have significant heterogeneity (*p* = 0.03, *I* ^*2*^ = 63%). The random-effects model was used in the meta-analysis. Compared with the model group, BBR could remarkably reduce the liver tissue TG level [*SMD* = 1.96, 95% *CI* (1.01, 2.92), *p* < 0.0001] (Supplementary Figure S10B 2.4.2). Two studies with 40 Wistar rats have reported the liver tissue TG level after BBR treatment. Liver tissue TG was analyzed to have significant heterogeneity (*p* = 0.07, *I* ^*2*^ = 69%). The random-effects model was used in the meta-analysis. Compared with the model group, BBR could remarkably reduce the liver tissue TG level [*SMD* = 2.30, 95% *CI* (0.75, 3.85), *p* = 0.004] (Supplementary Figure S10B 2.4.3). Two studies with 24 db/db mice have reported the liver tissue TG level after BBR treatment for NAFLD/NASH. There was minor heterogeneity in liver tissue TG level (*p* = 0.30, *I* ^*2*^ = 8%). So, the fixed-effects model was chosen in the meta-analysis. Compared with the model group, the liver tissue TG level was remarkably different [*SMD* = 2.20, 95% *CI* (1.07, 3.33, *p* = 0.0001] (Supplementary Figure S10C 2.4.4).

Due to space constraints, the subgroup analysis for different animal breeds of additional related major indicators such as ALT, AST, HDL-C, LDL-C, and FBG is shown in Supplementary Figures S11–14.

#### Subgroup Analysis for Different Models

In the HFD model, 19 studies with 362 animals have reported the blood TC level after BBR treatment for NAFLD/NASH. Blood TC showed significant heterogeneity (*p* < 0.00001, *I* ^*2*^ = 83%). The random-effects model was used in the meta-analysis. BBR could observably reduce the blood TC level compared with the model group [*SMD* = 2.30, 95% *CI* (1.61, 2.98), *p* < 0.00001] (Supplementary Figure S15A 3.1.1). In the MCD model, two studies with 34 animals have reported the blood TC level after BBR treatment for NAFLD/NASH. Blood TC level was found to have apparent heterogeneity (*p* = 0.03, *I* ^*2*^ = 78%). Therefore, we used the fixed-effects model in the meta-analysis. However, there were no significance compared with the model group [*SMD* = 0.00, 95% *CI* (−1.51, 1.52), *p* = 1.00] (Supplementary Figure S15A 3.1.2).

In the HFD model, nine studies with 153 animals have reported the liver tissue TG level after BBR treatment for NAFLD/NASH. There was obvious heterogeneity in liver tissue TG level (*p* = 0.009, *I* ^*2*^ = 61%). So, the random-effects model was chosen in the meta-analysis. Compared with the model group, the liver tissue TG level was clearly different [*SMD* = 2.25, 95% *CI* (1.54, 2.96, *p* < 0.00001] (Supplementary Figure S15B). In the MCD model, two studies with 34 animals have reported the liver tissue TG level after BBR treatment. Liver tissue TG had minor heterogeneity (*p* = 0.76, *I* ^*2*^ = 0%). The fixed-effects model was used in the meta-analysis. Compared with the model group, BBR could remarkably reduce the liver tissue TG level [*SMD* = 1.55, 95% *CI* (0.75, 2.34), *p* = 0.0001] (Supplementary Figure S15C 3.2.2). In the db/db model, two studies with 24 animals have reported the liver tissue TG level after BBR treatment for NAFLD/NASH. There was minor heterogeneity in liver tissue TG level (*p* = 0.30, *I* ^*2*^ = 8%). Thus, we used the fixed-effects model in the meta-analysis. BBR could observably increase the liver tissue TG level compared with the model group [*SMD* = 2.20, 95% *CI* (1.07, 3.33), *p* = 0.0001] (Supplementary Figure S15C 3.2.3).

Due to space constraints, the subgroup analysis for different animal models of additional related major indicators such as ALT and AST is shown in Supplementary Figures S16–17.

### Publication Bias

In order to explore the publication bias and heterogeneity in-depth, we drew the funnel plots. As shown in Supplementary Figure S18, the funnel plots of main outcomes such as TC, TG, ALT, AST, HDL-C, LDL-C, FBG, FINS, HOMA-IR, and FFA were asymmetric, suggesting the potential publication bias and heterogeneity.

## Discussion

### Exploration of Preclinical Mechanism

NAFLD is a chronic liver disease closely associated with obesity, type 2 diabetes, and hyperlipidemia and is becoming a serious health problem worldwide (Jahn et al., 2019). The major feature of NAFLD in the early stage is the slow accumulation of fat in hepatocytes (namely steatosis), and it can gradually develop into NASH, which involves tissue injury, chronic liver inflammation, and fibrosis and eventually contributes to end-stage liver disorder, including liver cirrhosis and hepatoma (Zhang et al., 2016; Goldberg et al., 2017). Since no approved medication is currently available for NAFLD/NASH, this forces us to determine potential pharmacological targets and create future therapies. Moreover, it is universally acknowledged that no animal model can completely reproduce the pathological condition of human NASH. How to transform the obtained results into further clinical researches is an ongoing challenge. To this end, a basic and translational study is still indispensable; in the translational process, preclinical systematic evidence plays a crucial role.

Different animal models with diverse mechanisms play various roles in the pathogenesis of NASH. Fortunately, the studies we considered identified the efficacy of BBR based on different models and mechanisms. The vast majority of included researches employed HFD to construct the animal model of NAFLD/NASH. The model presents similar characteristics of NASH pathology and metabolic syndromes such as hyperlipidemia and obesity. However, this model generally develops less severe hepatic inflammation and fibrosis, limiting its application for the study (Mow et al., 2004; Santhekadur et al., 2018). In this review, the results showed that BBR could improve HFD-fed animals’ disease state and restore the lipids content, insulin resistance, and glucose metabolism to reduce liver damage.

Several included studies have reported the NASH model based on the MCD diet, a commonly used diet that contains moderate fat (10%) and considerable sucrose (40%) but is deficient in methionine and choline. It can generally produce the extensive liver histological phenotype of NASH within only a few weeks (Weltman et al., 1996; Anstee et al., 2006; Yamada et al., 2016). Moreover, the major reason for the rapid onset is that the impairment in hepatic very-low-density lipoprotein (VLDL) secretion causes disorder in hepatocyte lipid metabolism accompanied by oxidative stress injury (Weltman et al., 1996; Lee et al., 2015). Therefore, this model is more suitable for discussing the histology of advanced NASH and the mechanism of fibrosis and inflammation than the other models. However, the limitations of this model are that some metabolic changes such as significant body weight loss and lack of insulin resistance have considerable disparities with human NASH. The studies considered have demonstrated that BBR could effectively alleviate the lipids content of MCD-fed animals to relieve liver injury.

Several studies have used db/db mice to conduct the NASH model. This kind of mice carries a point mutation of point mutation, which leads to defective leptin signaling, resulting in defective leptin signaling. As leptin is in charge of controlling feeding behavior through enhancing satiety, these mice generally have abnormal hyperphagia and are obese, hyperglycemic, hyperinsulinemic, and diabetic (Hummel et al., 1966; Yang et al., 1997; Trak-Smayra et al., 2011; Ibrahim et al., 2016). The studies with db/db mice have indicated that BBR could significantly alleviate the accumulation of fat in hepatocytes and reduce liver tissue damage.

We can conclude that although different models have relative disparities with mechanisms and characteristics, they all represent some pathological phenotypes of NAFLD/NASH. The subgroup analyses of different animal models in this review have shown the breathtaking therapeutic effects of BBR, although it exerted different targets and mechanisms and displayed different efficacy strengths when faced with different models.

### Discussion of the Subgroup Analyses on Different Rat Breeds

In this study, BBR was used to treat the NAFLD/NASH model caused by different breeds of animals, among which the therapeutic effects were significantly proved in most of the results. Due to the limited number of studies, some studies such as blood TG and TC in Wistar rats demonstrated to have a treatment trend, but the results were not significantly different, needing more researches to further identification. In addition, one study has revealed anti-fatty liver action of BBR in an HFD-induced larval zebrafish model besides rats or mice model (Chen et al., 2019), although we did not include it due to the lack of sample size. At present, more extensive animal breed experiments are still required to make up the gap.

### Discussion of Heterogeneity on Effect of BBR

The emergence of heterogeneity not only deepens the content of this review but also leaves wider issues to be explored. The occurrence of heterogeneity is influenced by multi-factors, including experimental design, study subjects, intervention measures, and results combination (Thompson et al., 1997; Hooijmans et al., 2014; Shi et al., 2021). The great heterogeneity difference in this review may be due to BBR and animal diets coming from different laboratories. The dosage of BBR varies between different studies and so does the composition of animal diets. Another issue is the inconsistency of the outcomes in terms of TC, TG, HDL-C, and LDL-C. The measurement kits, animal feeding conditions, laboratory temperatures, and even operations by different operators can lead to potential heterogeneity. Due to sample limitations, we were unable to implement subgroup analysis on the factors mentioned above. In experimental design and intervention measures, we performed a subgroup analysis of different animal breeds and animal models. Through the aforementioned subgroup analysis, we hoped to control the heterogeneity as much as possible and broaden the comprehension of BBR in the treatment of NAFLD/NASH. Simultaneously, mechanisms and sensitivity to BBR vary from different models, influencing the administration time and dose. These all need further in-depth research.

### Strength

The application of system reviews is conducive to preclinical design and clinical reliability. Furthermore, it can contribute to conducting experimental animal research and eliminating unnecessary experiments so as to explore the potential value of BBR in treating hepatic diseases in-depth, enhance the dependability of future clinical trials, and promote the extensive application of BBR. Additionally, traditional Chinese medicine containing berberine has a long history of application in China, and berberine has been used in several over-the-counter (OTC) drugs to treat gastroenteritis, indicating its relative safety and efficacy (Vivoli et al., 2016).

**FIGURE 4:**
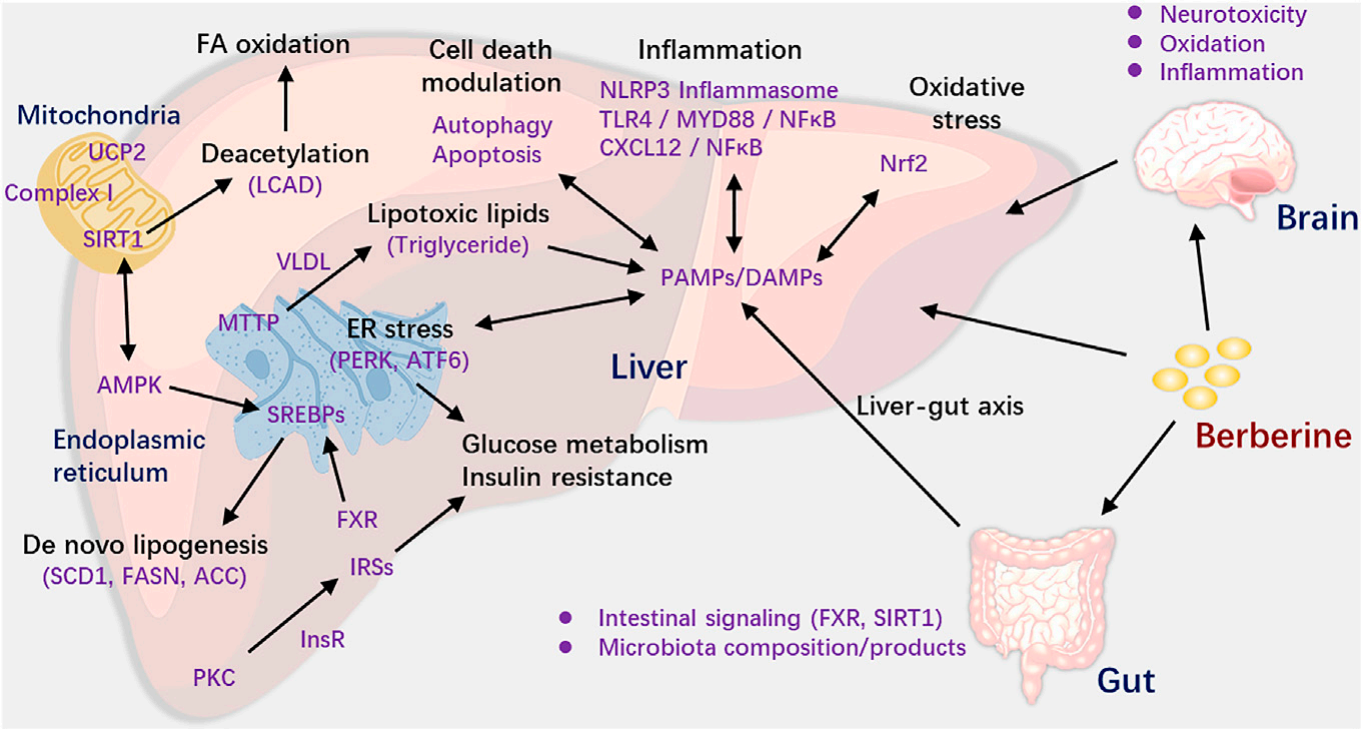
Role and mechanisms of BBR in treating NAFLD.

According to the results of this review, we can see that BBR was suitable for a range of NAFLD/NASH models. Based on the studies we included, we mapped the mechanism figure of berberine in the treatment of NAFLD/NASH. We could see the results from Figure 4 that BBR could activate the adenosine monophosphate-activated protein kinase (AMPK) pathway (Lee et al., 2006; Kim et al., 2009; Zhou et al., 2017), ameliorate insulin sensitivity and glucose metabolism (Lee et al., 2006; Chen et al., 2009; Kong et al., 2009), regulate mitochondrial function (Teodoro et al., 2013; Xu et al., 2014), alleviate inflammation and oxidative stress (Vivoli et al., 2016; Dinesh et al., 2017; Mahmoud et al., 2017; Zhou et al., 2017), modulate cell death and ER stress (He et al., 2016; Zhou et al., 2017; Mai et al., 2020), reduce DNA methylation (Chang et al., 2010), and regulate gut microenvironment (Zhang et al., 2012; Zhang Y. et al., 2015) and neurotoxicity (Kim et al., 2009; Ghareeb et al., 2015). Although in different models, the action mode of BBR is different, BBR could reduce the levels of TC, TG, ALT, AST, and so forth via the above mechanisms and in turn ameliorate the liver function.

Moreover, the effect of BBR varies among the different animal breeds and based on the administration time. Hence, in the experimental design, different species and administration times were applied according to different experimental purposes. Besides, more complicated issues, such as the dose range of BBR, are supposed to be given more focus. The overall results suggest that berberine plays a vital role in NAFLD/NASH. Further research on the related mechanism is warranted.

### Limitations

We took into account the internal and external factors, including room temperature, laboratory equipment, BBR dosage, and different selection of kits in this previous review. Nevertheless, the researches with a detailed description in terms of their methodological procedures and transparency are not abundant. Thus, controlling the sources of the potential heterogeneity will be the focus of future studies. Furthermore, the asymmetric funnel plots suggested potential publication bias, which might result from the following causes. On the one hand, the researches were conducted in different laboratories, some of which were not GLP centers. On the other hand, the animal sizes varied from 4 to 20. The publication bias may be a possible interpretation for such a small-study effects bias. Therefore, the entire experiment process and limited animal sizes might contribute meaningfully to the increment of variability and potential bias.

In NAFLD basic research, many animal researchers are prone to regard blinding and randomization as redundant. In fact, some of the studies we included had a similar concept as the authors generally believed that their animals come from a sample group with homogeneous genetic background and environment. However, not only the animals but also the inducement of NAFLD may cause alteration, thereby weakening the rigor. Secondly, if each animal represents almost the same or unique sample, then repeating the experiment on such a sample will produce erroneous results. Considering that randomization is simple, practical, and cost-effective, it should be recommended to be an essential content of the experiment.

Additionally, no data in this study reported the calculation of the absorption dosage of BBR. That may be because the determination of BBR absorption is still technically difficult. Simultaneously, it is also challenging for us to unify the degree of absorption of different methods, such as gavage and intraperitoneal injection of BBR. Last but not least, no studies have taken into account gender and age differences in terms of efficacy, because all studies used adult animals aged 4–9 weeks without young or old animals and all studies except one (Ghareeb et al., 2015) have used male animals, which need pay more attention in further exploration. In conclusion, the results are supposed to be interpreted with reason and caution.

## Conclusion

In a nutshell, this systematic preclinical review demonstrated that BBR could effectively reduce the body weight, liver weight, NAS, steatosis score, and other disease characteristics. From the analysis of the mechanism, BBR could remarkably alleviate the lipids content, improve insulin resistance glucose metabolism, and anti-inflammatory, anti-oxidative stress, and in turn ameliorate the liver function. The subgroup analysis of animal breeds and models further indicated features of BBR on NAFLD/NASH. Therefore, BBR is suggested to have a unique effect on NAFLD/NASH from the current documents. In the future, the conclusion should be interpreted with reason and caution based on more well-designed experiments.

## Data Availability

The original contributions presented in the study are included in the article/Supplementary Material; further inquiries can be directed to the corresponding author.

## Abbreviations

BBR: berberine; NAFLD: non-alcoholic fatty liver disease; NASH: non-alcoholic fatty liver disease; TC: total cholesterol; TG: triglycerides; ALT: alanine aminotransferase; AST: aspartate amino-transferase, HDL-C: high-density lipoprotein cholesterol; LDL-C: low-density lipoprotein cholesterol; FBG: fasting blood glucose; FINS: fasting insulin; HOMA-IR: homeostasis model assessment-insulin resistance; FFA: free fat acid; NAS: NAFLD activity score; TNF-α: tumor necrosis factor-α; IL-6: interleukin-6; IL-1β: interleukin-1β; GSH: glutathione; TBARS: thiobarbituric acid reactive substances; SD: Sprague–Dawley; dbdb: C57BLKS/J-Lepr^db^/Lepr^db;^ HFD: high-fat diet; MCD: methionine- and choline-deficient; TM: tunicamycin; SMD: std. mean difference; CI: confidence interval.

## References

[B1] Angulo P. (2007). GI Epidemiology: Nonalcoholic Fatty Liver Disease. Aliment. Pharmacol. Ther. 25 (8), 883–889. 10.1111/j.1365-2036.2007.03246.x 17402991

[B2] Anstee Q. M. Goldin R. D. (2006). Mouse Models in Non-alcoholic Fatty Liver Disease and Steatohepatitis Research. Int. J. Exp. Pathol. 87 (1), 1–16. 10.1111/j.0959-9673.2006.00465.x 16436109 PMC2517349

[B3] Cao Y. Pan Q. Cai W. Shen F. Chen G. Y. Xu L. M. (2016). Modulation of Gut Microbiota by Berberine Improves Steatohepatitis in High-Fat Diet-Fed BALB/C Mice. Arch. Iran Med. 19 (3), 197–203. 10.0161903/AIM.008 26923892

[B4] Chang X. Yan H. Fei J. Jiang M. Zhu H. Lu D. (2010). Berberine Reduces Methylation of the MTTP Promoter and Alleviates Fatty Liver Induced by a High-Fat Diet in Rats. J. Lipid Res. 51 (9), 2504–2515. 10.1194/jlr.M001958 20567026 PMC2918435

[B5] Chen B. Zheng Y. M. Zhang M. Q. Han Y. Zhang J. P. Hu C. Q. (2019). Microarray Expression Profiling and Raman Spectroscopy Reveal Anti-fatty Liver Action of Berberine in a Diet-Induced Larval Zebrafish Model. Front. Pharmacol. 10, 1504. 10.3389/fphar.2019.01504 31969822 PMC6960226

[B6] Chen Y. Li Y. Wang Y. Wen Y. Sun C. (2009). Berberine Improves Free-Fatty-Acid-Induced Insulin Resistance in L6 Myotubes through Inhibiting Peroxisome Proliferator-Activated Receptor Gamma and Fatty Acid Transferase Expressions. Metabolism 58 (12), 1694–1702. 10.1016/j.metabol.2009.06.009 19767038

[B7] Day C. P. James O. F. (1998). Steatohepatitis: a Tale of Two "hits"? Gastroenterology 114 (4), 842–845. 10.1016/s0016-5085(98)70599-2 9547102

[B8] Deng Y. Tang K. Chen R. Nie H. Liang S. Zhang J. (2019). Berberine Attenuates Hepatic Oxidative Stress in Rats with Non-alcoholic Fatty Liver Disease via the Nrf2/ARE Signalling Pathway. Exp. Ther. Med. 17 (3), 2091–2098. 10.3892/etm.2019.7208 30867696 PMC6396022

[B9] Dinesh P. Rasool M. (2017). Berberine, an Isoquinoline Alkaloid Suppresses TXNIP Mediated NLRP3 Inflammasome Activation in MSU crystal Stimulated RAW 264.7 Macrophages through the Upregulation of Nrf2 Transcription Factor and Alleviates MSU crystal Induced Inflammation in Rats. Int. Immunopharmacol 44, 26–37. 10.1016/j.intimp.2016.12.031 28068647

[B10] Eman E. Hemat E. Hanaa H. Safwat Q. (2019). Effects of Berberine on High-Fat/high-Sucrose-Induced Nonalcoholic Steatohepatitis in Experimental Rats. Tanta Med. J. 47 (2), 80. 10.4103/tmj.tmj_15_18

[B11] Fan J. G. Farrell G. C. (2009). Epidemiology of Non-alcoholic Fatty Liver Disease in China. J. Hepatol. 50 (1), 204–210. 10.1016/j.jhep.2008.10.010 19014878

[B12] Feng W. W. Kuang S. Y. Tu C. Ma Z. J. Pang J. Y. Wang Y. H. (2018). Natural Products Berberine and Curcumin Exhibited Better Ameliorative Effects on Rats with Non-alcohol Fatty Liver Disease Than Lovastatin. Biomed. Pharmacother. 99, 325–333. 10.1016/j.biopha.2018.01.071 29353208

[B13] Ghareeb D. A. Khalil S. Hafez H. S. Bajorath J. Ahmed H. E. Sarhan E. (2015). Berberine Reduces Neurotoxicity Related to Nonalcoholic Steatohepatitis in Rats. Evid. Based Complement. Alternat Med. 2015, 361847. 10.1155/2015/361847 26576191 PMC4630388

[B14] Goldberg D. Ditah I. C. Saeian K. Lalehzari M. Aronsohn A. Gorospe E. C. (2017). Changes in the Prevalence of Hepatitis C Virus Infection, Nonalcoholic Steatohepatitis, and Alcoholic Liver Disease Among Patients with Cirrhosis or Liver Failure on the Waitlist for Liver Transplantation. Gastroenterology 152 (5), 1090–e1091. 10.1053/j.gastro.2017.01.003 28088461 PMC5367965

[B15] He Q. Mei D. Sha S. Fan S. Wang L. Dong M. (2016). ERK-dependent mTOR Pathway Is Involved in Berberine-Induced Autophagy in Hepatic Steatosis. J. Mol. Endocrinol. 57 (4), 251–260. 10.1530/jme-16-0139 27658958

[B16] Hooijmans C. R. Rovers M. M. de Vries R. B. Leenaars M. Ritskes-Hoitinga M. Langendam M. W. (2014). SYRCLE's Risk of Bias Tool for Animal Studies. BMC Med. Res. Methodol. 14, 43. 10.1186/1471-2288-14-43 24667063 PMC4230647

[B17] Hummel K. P. Dickie M. M. Coleman D. L. (1966). Diabetes, a New Mutation in the Mouse. Science 153 (3740), 1127–1128. 10.1126/science.153.3740.1127 5918576

[B18] Ibrahim S. H. Hirsova P. Malhi H. Gores G. J. (2016). Animal Models of Nonalcoholic Steatohepatitis: Eat, Delete, and Inflame. Dig. Dis. Sci. 61 (5), 1325–1336. 10.1007/s10620-015-3977-1 26626909 PMC4838538

[B19] Jahn D. Kircher S. Hermanns H. M. Geier A. (2019). Animal Models of NAFLD from a Hepatologist's point of View. Biochim. Biophys. Acta Mol. Basis Dis. 1865 (5), 943–953. 10.1016/j.bbadis.2018.06.023 29990551

[B20] Kim W. S. Lee Y. S. Cha S. H. Jeong H. W. Choe S. S. Lee M. R. (2009). Berberine Improves Lipid Dysregulation in Obesity by Controlling central and Peripheral AMPK Activity. Am. J. Physiol. Endocrinol. Metab. 296 (4), E812–E819. 10.1152/ajpendo.90710.2008 19176354

[B21] Kong W. Wei J. Abidi P. Lin M. Inaba S. Li C. (2004). Berberine Is a Novel Cholesterol-Lowering Drug Working through a Unique Mechanism Distinct from Statins. Nat. Med. 10 (12), 1344–1351. 10.1038/nm1135 15531889

[B22] Kong W. J. Zhang H. Song D. Q. Xue R. Zhao W. Wei J. (2009). Berberine Reduces Insulin Resistance through Protein Kinase C-dependent Up-Regulation of Insulin Receptor Expression. Metabolism 58 (1), 109–119. 10.1016/j.metabol.2008.08.013 19059538

[B23] Lee S. J. Kang J. H. Iqbal W. Kwon O. S. (2015). Proteomic Analysis of Mice Fed Methionine and Choline Deficient Diet Reveals Marker Proteins Associated with Steatohepatitis. PLoS One 10 (4), e0120577. 10.1371/journal.pone.0120577 25849376 PMC4388516

[B24] Lee Y. S. Kim W. S. Kim K. H. Yoon M. J. Cho H. J. Shen Y. (2006). Berberine, a Natural Plant Product, Activates AMP-Activated Protein Kinase with Beneficial Metabolic Effects in Diabetic and Insulin-Resistant States. Diabetes 55 (8), 2256–2264. 10.2337/db06-0006 16873688

[B25] Li D. Zheng J. Hu Y. Hou H. Hao S. Liu N. (2017). Amelioration of Intestinal Barrier Dysfunction by Berberine in the Treatment of Nonalcoholic Fatty Liver Disease in Rats. Pharmacogn Mag. 13 (52), 677–682. 10.4103/pm.pm_584_16 29200733 PMC5701411

[B26] Li J. Liu Z. Guo M. Xu K. Jiang M. Lu A. (2015). Metabolomics Profiling to Investigate the Pharmacologic Mechanisms of Berberine for the Treatment of High-Fat Diet-Induced Nonalcoholic Steatohepatitis. Evid. Based Complement. Alternat Med. 2015, 897914. 10.1155/2015/897914 25977701 PMC4421035

[B27] Liang H. Wang Y. (2018). Berberine Alleviates Hepatic Lipid Accumulation by Increasing ABCA1 through the Protein Kinase C δ Pathway. Biochem. Biophys. Res. Commun. 498 (3), 473–480. 10.1016/j.bbrc.2018.03.003 29505790

[B28] Lu Z. He B. Chen Z. Yan M. Wu L. (2020). Anti-inflammatory Activity of Berberine in Non-alcoholic Fatty Liver Disease via the Angptl2 Pathway. BMC Immunol. 21 (1), 28. 10.1186/s12865-020-00358-9 32429849 PMC7236478

[B29] Lu Z. Lu F. Wu L. He B. Chen Z. Yan M. (2021). Berberine Attenuates Non-alcoholic Steatohepatitis by Regulating chemerin/CMKLR1 Signalling Pathway and Treg/Th17 Ratio. Naunyn Schmiedebergs Arch. Pharmacol. 394 (2), 383–390. 10.1007/s00210-020-01914-1 32524150

[B30] Mahmoud A. M. Hozayen W. G. Ramadan S. M. (2017). Berberine Ameliorates Methotrexate-Induced Liver Injury by Activating Nrf2/HO-1 Pathway and PPARγ, and Suppressing Oxidative Stress and Apoptosis in Rats. Biomed. Pharmacother. 94, 280–291. 10.1016/j.biopha.2017.07.101 28763751

[B31] Mai W. Xu Y. Xu J. Zhao D. Ye L. Yu G. (2020). Berberine Inhibits Nod-like Receptor Family Pyrin Domain Containing 3 Inflammasome Activation and Pyroptosis in Nonalcoholic Steatohepatitis via the ROS/TXNIP Axis. Front. Pharmacol. 11, 185. 10.3389/fphar.2020.00185 32194416 PMC7063468

[B32] Malaguarnera M. Di Rosa M. Nicoletti F. Malaguarnera L. (2009). Molecular Mechanisms Involved in NAFLD Progression. J. Mol. Med. (Berl) 87 (7), 679–695. 10.1007/s00109-009-0464-1 19352614

[B33] Mo C. Wang L. Zhang J. Numazawa S. Tang H. Tang X. (2014). The Crosstalk between Nrf2 and AMPK Signal Pathways Is Important for the Anti-inflammatory Effect of Berberine in LPS-Stimulated Macrophages and Endotoxin-Shocked Mice. Antioxid. Redox Signal. 20 (4), 574–588. 10.1089/ars.2012.5116 23875776 PMC3901384

[B34] Mow W. S. Vasiliauskas E. A. Lin Y. C. Fleshner P. R. Papadakis K. A. Taylor K. D. (2004). Association of Antibody Responses to Microbial Antigens and Complications of Small Bowel Crohn's Disease. Gastroenterology 126 (2), 414–424. 10.1053/j.gastro.2003.11.015 14762777

[B35] Peters J. L. Sutton A. J. Jones D. R. Rushton L. Abrams K. R. (2006). A Systematic Review of Systematic Reviews and Meta-Analyses of Animal Experiments with Guidelines for Reporting. J. Environ. Sci. Health B 41 (7), 1245–1258. 10.1080/03601230600857130 16923604

[B36] Ragab S. M. Abd Elghaffar S. Kh. El-Metwally T. H. Badr G. Mahmoud M. H. Omar H. M. (2015). Effect of a High Fat, High Sucrose Diet on the Promotion of Non-alcoholic Fatty Liver Disease in Male Rats: the Ameliorative Role of Three Natural Compounds. Lipids Health Dis. 14, 83. 10.1186/s12944-015-0087-1 26228038 PMC4520282

[B37] Santhekadur P. K. Kumar D. P. Sanyal A. J. (2018). Preclinical Models of Non-alcoholic Fatty Liver Disease. J. Hepatol. 68 (2), 230–237. 10.1016/j.jhep.2017.10.031 29128391 PMC5775040

[B38] Scherer A. Dufour J. F. (2016). Treatment of Non-alcoholic Fatty Liver Disease. Dig. Dis. 34 (Suppl. 1), 27–31. 10.1159/000447278 27548081

[B39] Shi K. Wen J. Zeng J. Guo Y. Hu J. Li C. (2021). Preclinical Evidence of Yinchenhao Decoction on Cholestasis: A Systematic Review and Meta-Analysis of Animal Studies. Phytother Res. 35 (1), 138–154. 10.1002/ptr.6806 32975338

[B40] Sumida Y. Yoneda M. (2018). Current and Future Pharmacological Therapies for NAFLD/NASH. J. Gastroenterol. 53 (3), 362–376. 10.1007/s00535-017-1415-1 29247356 PMC5847174

[B41] Sun R. Yang N. Kong B. Cao B. Feng D. Yu X. (2017). Orally Administered Berberine Modulates Hepatic Lipid Metabolism by Altering Microbial Bile Acid Metabolism and the Intestinal FXR Signaling Pathway. Mol. Pharmacol. 91 (2), 110–122. 10.1124/mol.116.106617 27932556 PMC5267522

[B42] Sun Y. Xia M. Yan H. Han Y. Zhang F. Hu Z. (2018). Berberine Attenuates Hepatic Steatosis and Enhances Energy Expenditure in Mice by Inducing Autophagy and Fibroblast Growth Factor 21. Br. J. Pharmacol. 175 (2), 374–387. 10.1111/bph.14079 29065221 PMC5758394

[B43] Teodoro J. S. Duarte F. V. Gomes A. P. Varela A. T. Peixoto F. M. Rolo A. P. (2013). Berberine Reverts Hepatic Mitochondrial Dysfunction in High-Fat Fed Rats: A Possible Role for SirT3 Activation. Mitochondrion 13 (6), 637–646. 10.1016/j.mito.2013.09.002 24041461

[B44] Thompson S. G. Smith T. C. Sharp S. J. (1997). Investigating Underlying Risk as a Source of Heterogeneity in Meta-Analysis. Stat. Med. 16 (23), 2741–2758. 10.1002/(sici)1097-0258(19971215)16:23<2741::aid-sim703>3.0.co;2-0 9421873

[B45] Tilg H. Moschen A. R. (2010). Evolution of Inflammation in Nonalcoholic Fatty Liver Disease: the Multiple Parallel Hits Hypothesis. Hepatology 52 (5), 1836–1846. 10.1002/hep.24001 21038418

[B46] Tillhon M. Guamán Ortiz L. M. Lombardi P. Scovassi A. I. (2012). Berberine: New Perspectives for Old Remedies. Biochem. Pharmacol. 84 (10), 1260–1267. 10.1016/j.bcp.2012.07.018 22842630

[B47] Trak-Smayra V. Paradis V. Massart J. Nasser S. Jebara V. Fromenty B. (2011). Pathology of the Liver in Obese and Diabetic Ob/ob and Db/db Mice Fed a Standard or High-Calorie Diet. Int. J. Exp. Pathol. 92 (6), 413–421. 10.1111/j.1365-2613.2011.00793.x 22118645 PMC3248077

[B48] Vivoli E. Cappon A. Milani S. Piombanti B. Provenzano A. Novo E. (2016). NLRP3 Inflammasome as a Target of Berberine in Experimental Murine Liver Injury: Interference with P2X7 Signalling. Clin. Sci. (Lond) 130 (20), 1793–1806. 10.1042/CS20160400 27439970

[B49] Wang L. Jia Z. Wang B. Zhang B. (2020). Berberine Inhibits Liver Damage in Rats with Non-alcoholic Fatty Liver Disease by Regulating TLR4/MyD88/NF-Κb Pathway. Turk J. Gastroenterol. 31 (12), 902–909. 10.5152/tjg.2020.19568 33626003 PMC7928262

[B50] Wang Y. Campbell T. Perry B. Beaurepaire C. Qin L. (2011). Hypoglycemic and Insulin-Sensitizing Effects of Berberine in High-Fat Diet- and Streptozotocin-Induced Diabetic Rats. Metabolism 60 (2), 298–305. 10.1016/j.metabol.2010.02.005 20304443

[B51] Wang Y. Cui S. Zheng J. Li Y. Li P. Hou H. (2020). Berberine Ameliorates Intestinal Mucosal Barrier Dysfunction in Nonalcoholic Fatty Liver Disease (NAFLD) Rats. J. King Saud Univ. - Sci. 32 (5), 2534–2539. 10.1016/j.jksus.2020.03.019

[B52] Weltman M. D. Farrell G. C. Liddle C. (1996). Increased Hepatocyte CYP2E1 Expression in a Rat Nutritional Model of Hepatic Steatosis with Inflammation. Gastroenterology 111 (6), 1645–1653. 10.1016/s0016-5085(96)70028-8 8942745

[B53] Williams R. (2006). Global Challenges in Liver Disease. Hepatology 44 (3), 521–526. 10.1002/hep.21347 16941687

[B54] Wong R. J. Cheung R. Ahmed A. (2014). Nonalcoholic Steatohepatitis Is the Most Rapidly Growing Indication for Liver Transplantation in Patients with Hepatocellular Carcinoma in the U.S. Hepatology 59 (6), 2188–2195. 10.1002/hep.26986 24375711

[B55] Xing L. J. Zhang L. Liu T. Hua Y. Q. Zheng P. Y. Ji G. (2011). Berberine Reducing Insulin Resistance by Up-Regulating IRS-2 mRNA Expression in Nonalcoholic Fatty Liver Disease (NAFLD) Rat Liver. Eur. J. Pharmacol. 668 (3), 467–471. 10.1016/j.ejphar.2011.07.036 21839075

[B56] Xu M. Xiao Y. Yin J. Hou W. Yu X. Shen L. (2014). Berberine Promotes Glucose Consumption Independently of AMP-Activated Protein Kinase Activation. PLoS One 9 (7), e103702. 10.1371/journal.pone.0103702 25072399 PMC4114874

[B57] Xu X. Zhu X. P. Bai J. Y. Xia P. Li Y. Lu Y. (2019). Berberine Alleviates Nonalcoholic Fatty Liver Induced by a High-Fat Diet in Mice by Activating SIRT3. FASEB J. 33 (6), 7289–7300. 10.1096/fj.201802316R 30848932

[B58] Yamada T. Obata A. Kashiwagi Y. Rokugawa T. Matsushima S. Hamada T. (2016). Gd-EOB-DTPA-enhanced-MR Imaging in the Inflammation Stage of Nonalcoholic Steatohepatitis (NASH) in Mice. Magn. Reson. Imaging 34 (6), 724–729. 10.1016/j.mri.2016.03.009 26979540

[B59] Yang J. Ma X. J. Li L. Wang L. Chen Y. G. Liu J. (2017). Berberine Ameliorates Non-alcoholic Steatohepatitis in ApoE-/- Mice. Exp. Ther. Med. 14 (5), 4134–4140. 10.3892/etm.2017.5051 29075339 PMC5647746

[B60] Yang Q. H. Hu S. P. Zhang Y. P. Xie W. N. Li N. Ji G. Y. (2011). Effect of Berberine on Expressions of Uncoupling Protein-2 mRNA and Protein in Hepatic Tissue of Non-alcoholic Fatty Liver Disease in Rats. Chin. J. Integr. Med. 17 (3), 205–211. 10.1007/s11655-011-0668-4 21359922

[B61] Yang S. Q. Lin H. Z. Lane M. D. Clemens M. Diehl A. M. (1997). Obesity Increases Sensitivity to Endotoxin Liver Injury: Implications for the Pathogenesis of Steatohepatitis. Proc. Natl. Acad. Sci. U S A. 94 (6), 2557–2562. 10.1073/pnas.94.6.2557 9122234 PMC20127

[B62] Yu M. Alimujiang M. Hu L. Liu F. Bao Y. Yin J. (2021). Berberine Alleviates Lipid Metabolism Disorders via Inhibition of Mitochondrial Complex I in Gut and Liver. Int. J. Biol. Sci. 17 (7), 1693–1707. 10.7150/ijbs.54604 33994854 PMC8120465

[B63] Yuan X. Wang J. Tang X. Li Y. Xia P. Gao X. (2015). Berberine Ameliorates Nonalcoholic Fatty Liver Disease by a Global Modulation of Hepatic mRNA and lncRNA Expression Profiles. J. Transl Med. 13, 24. 10.1186/s12967-015-0383-6 25623289 PMC4316752

[B64] Zhang X. Zhao Y. Xu J. Xue Z. Zhang M. Pang X. (2015a). Modulation of Gut Microbiota by Berberine and Metformin during the Treatment of High-Fat Diet-Induced Obesity in Rats. Sci. Rep. 5, 14405. 10.1038/srep14405 26396057 PMC4585776

[B65] Zhang X. Zhao Y. Zhang M. Pang X. Xu J. Kang C. (2012). Structural Changes of Gut Microbiota during Berberine-Mediated Prevention of Obesity and Insulin Resistance in High-Fat Diet-Fed Rats. PLoS One 7 (8), e42529. 10.1371/journal.pone.0042529 22880019 PMC3411811

[B66] Zhang Y. Chang X. Song X. Chen C. Chen H. Lu Z. (2015b). Berberine Reverses Abnormal Expression of L-type Pyruvate Kinase by DNA Demethylation and Histone Acetylation in the Livers of the Non-alcoholic Fatty Disease Rat. Int. J. Clin. Exp. Med. 8 (5), 7535–7543. 26221297 PMC4509242

[B67] Zhang Y. P. Deng Y. J. Tang K. R. Chen R. S. Liang S. Liang Y. J. (2019). Berberine Ameliorates High-Fat Diet-Induced Non-alcoholic Fatty Liver Disease in Rats via Activation of SIRT3/AMPK/ACC Pathway. Curr. Med. Sci. 39 (1), 37–43. 10.1007/s11596-019-1997-3 30868489

[B68] Zhang Z. Li B. Meng X. Yao S. Jin L. Yang J. (2016). Berberine Prevents Progression from Hepatic Steatosis to Steatohepatitis and Fibrosis by Reducing Endoplasmic Reticulum Stress. Sci. Rep. 6, 20848. 10.1038/srep20848 26857750 PMC4746620

[B69] Zhao H. L. Sui Y. Qiao C. F. Yip K. Y. Leung R. K. Tsui S. K. (2012). Sustained Antidiabetic Effects of a Berberine-Containing Chinese Herbal Medicine through Regulation of Hepatic Gene Expression. Diabetes 61 (4), 933–943. 10.2337/db11-1164 22396199 PMC3314348

[B70] Zhao J. Wang Y. Wu X. Tong P. Yue Y. Gao S. (2018). Inhibition of CCL19 Benefits Non alcoholic Fatty Liver Disease by Inhibiting TLR4/NFκBp65 Signaling. Mol. Med. Rep. 18 (5), 4635–4642. 10.3892/mmr.2018.9490 30221732

[B71] Zhao L. Cang Z. Sun H. Nie X. Wang N. Lu Y. (2017). Berberine Improves Glucogenesis and Lipid Metabolism in Nonalcoholic Fatty Liver Disease. BMC Endocr. Disord. 17 (1), 13. 10.1186/s12902-017-0165-7 28241817 PMC5329945

[B72] Zhou H. Feng L. Xu F. Sun Y. Ma Y. Zhang X. (2017). Berberine Inhibits Palmitate-Induced NLRP3 Inflammasome Activation by Triggering Autophagy in Macrophages: A New Mechanism Linking Berberine to Insulin Resistance Improvement. Biomed. Pharmacother. 89, 864–874. 10.1016/j.biopha.2017.03.003 28282788

[B73] Zhu X. Bian H. Wang L. Sun X. Xu X. Yan H. (2019). Berberine Attenuates Nonalcoholic Hepatic Steatosis through the AMPK-SREBP-1c-SCD1 Pathway. Free Radic. Biol. Med. 141, 192–204. 10.1016/j.freeradbiomed.2019.06.019 31226399

