# Supplementary material for: Preclinical Evidence of Berberine on Non-Alcoholic Fatty Liver Disease: A Systematic Review and Meta-Analysis of Animal Studies
Source: Front Pharmacol. 2021 Sep 9;12:742465. doi: 10.3389/fphar.2021.742465 (PMC8458904; doi:10.3389/fphar.2021.742465)
Supplement: Supplementary file 1 [file DataSheet1.doc]

**Supplementary Figures**


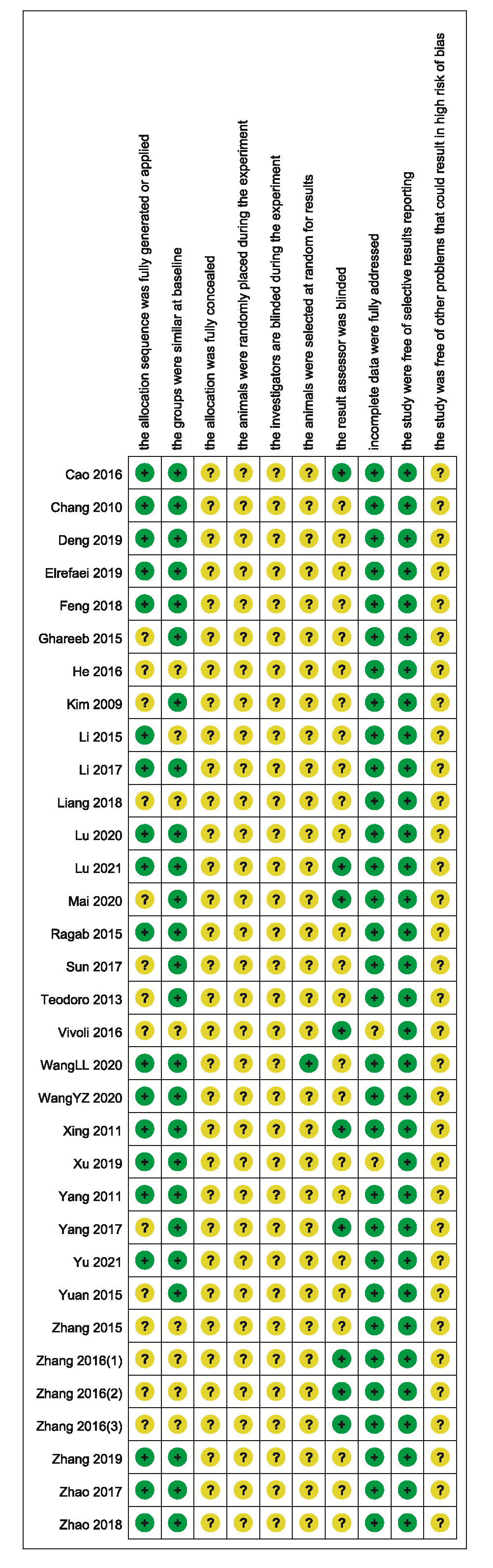


**Supplementary Figure 1.** **Risk of bias summary of included studies.**


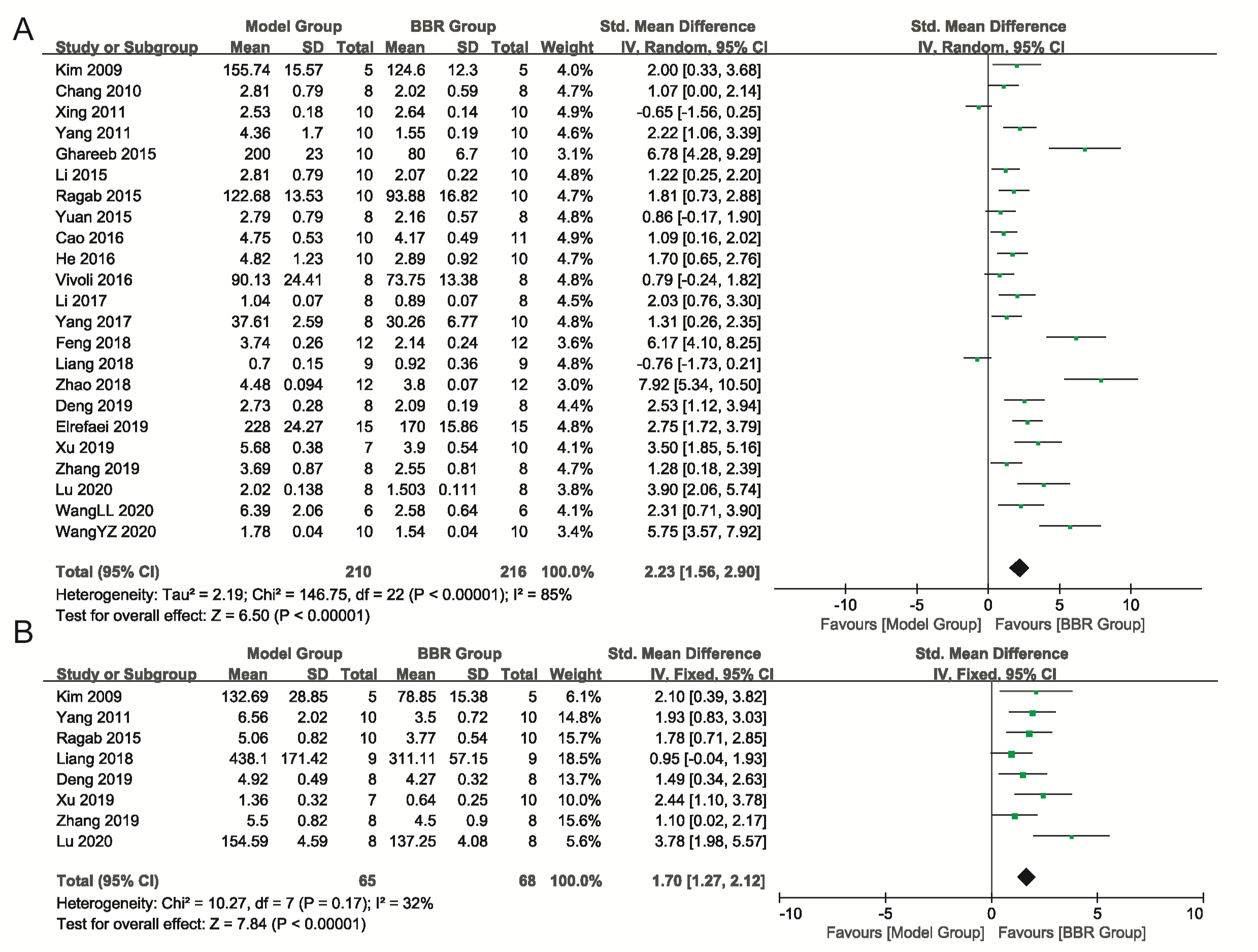


**Supplementary Figure 2. Forest plot of comparison.** (A) Blood TC; (B) Liver tissue TC; *I^2^* and *P* were taken as the heterogeneity test of the criterion. TC: total cholesterol.


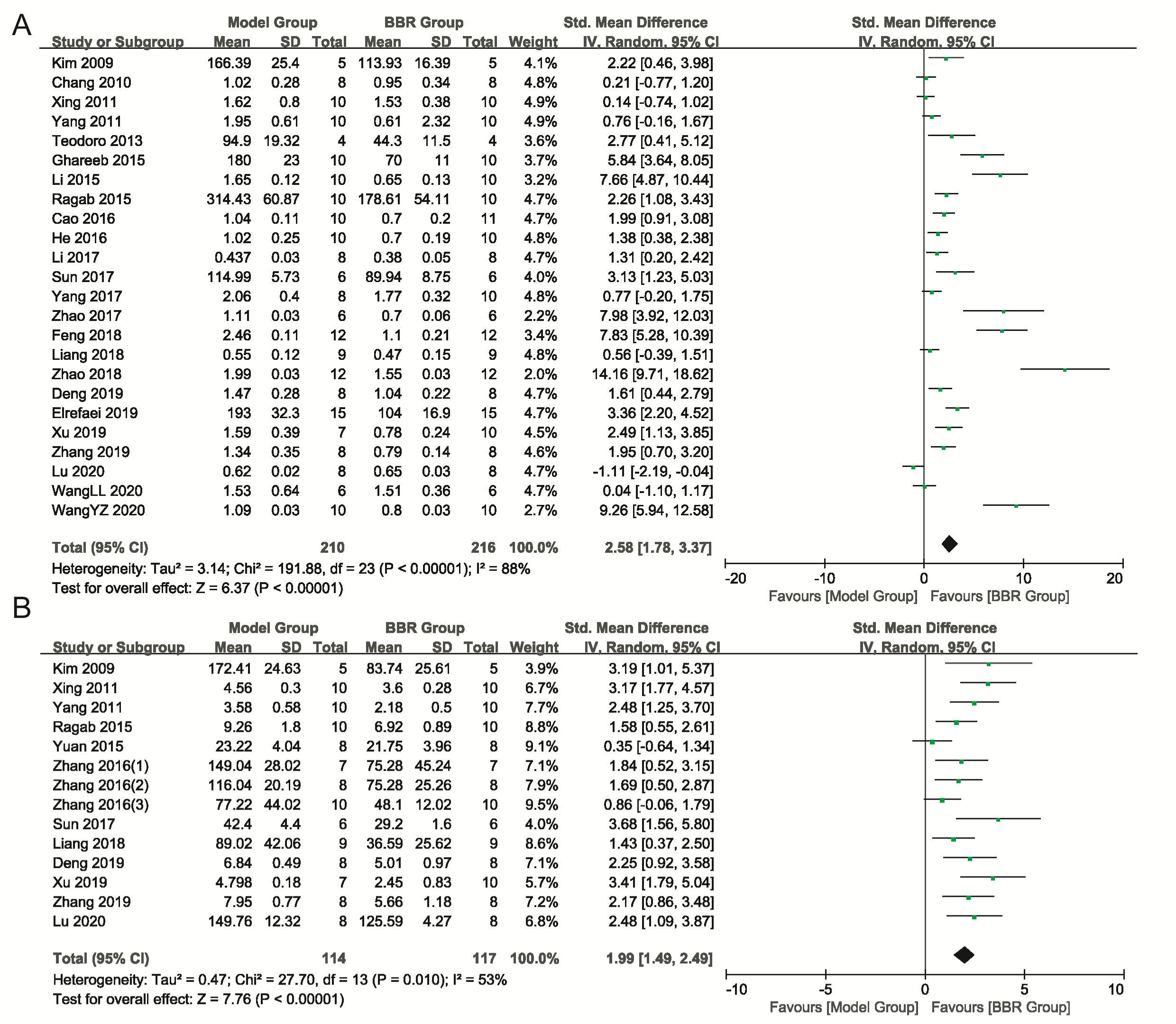


**Supplementary Figure 3. Forest plot of comparison.** (A) Blood TG; (B) Liver tissue TG; *I^2^* and *P* were taken as the heterogeneity test of the criterion. TG: triglycerides.


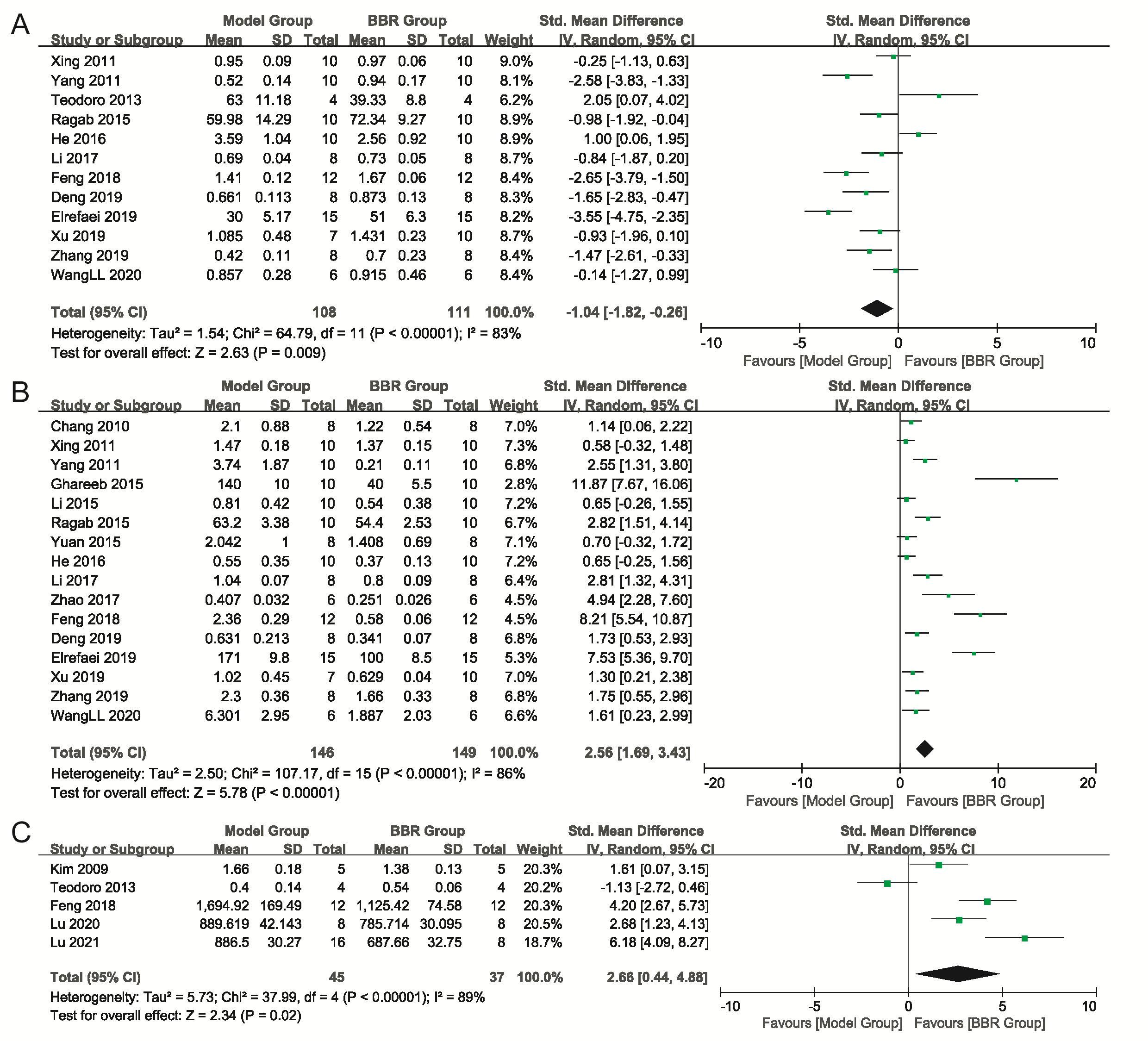


**Supplementary Figure 4. Forest plot of comparison.** (A) HDL-C; (B) LDL-C; (C) FFA; *I^2^* and *P* were taken as the heterogeneity test of the criterion. HDL-C: high-density lipoprotein cholesterol; LDL-C: low-density lipoprotein cholesterol; FFA: free fat acid.


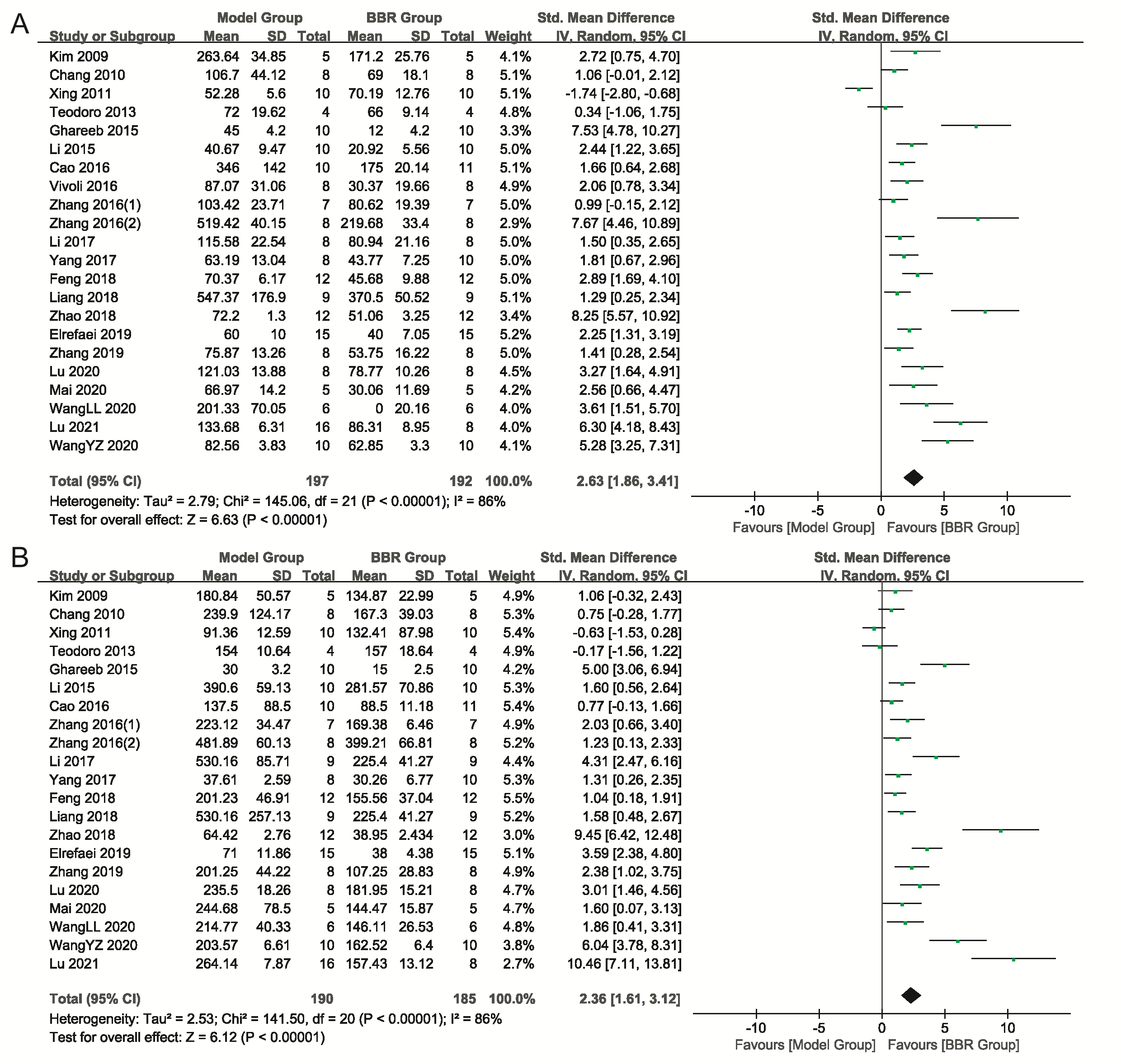
**Supplementary Figure 5. Forest plot of comparison.** (A) ALT; (B) AST; *I^2^* and *P* were taken as the heterogeneity test of the criterion. ALT: alanine aminotransferase; AST: aspartate amino-transferase.


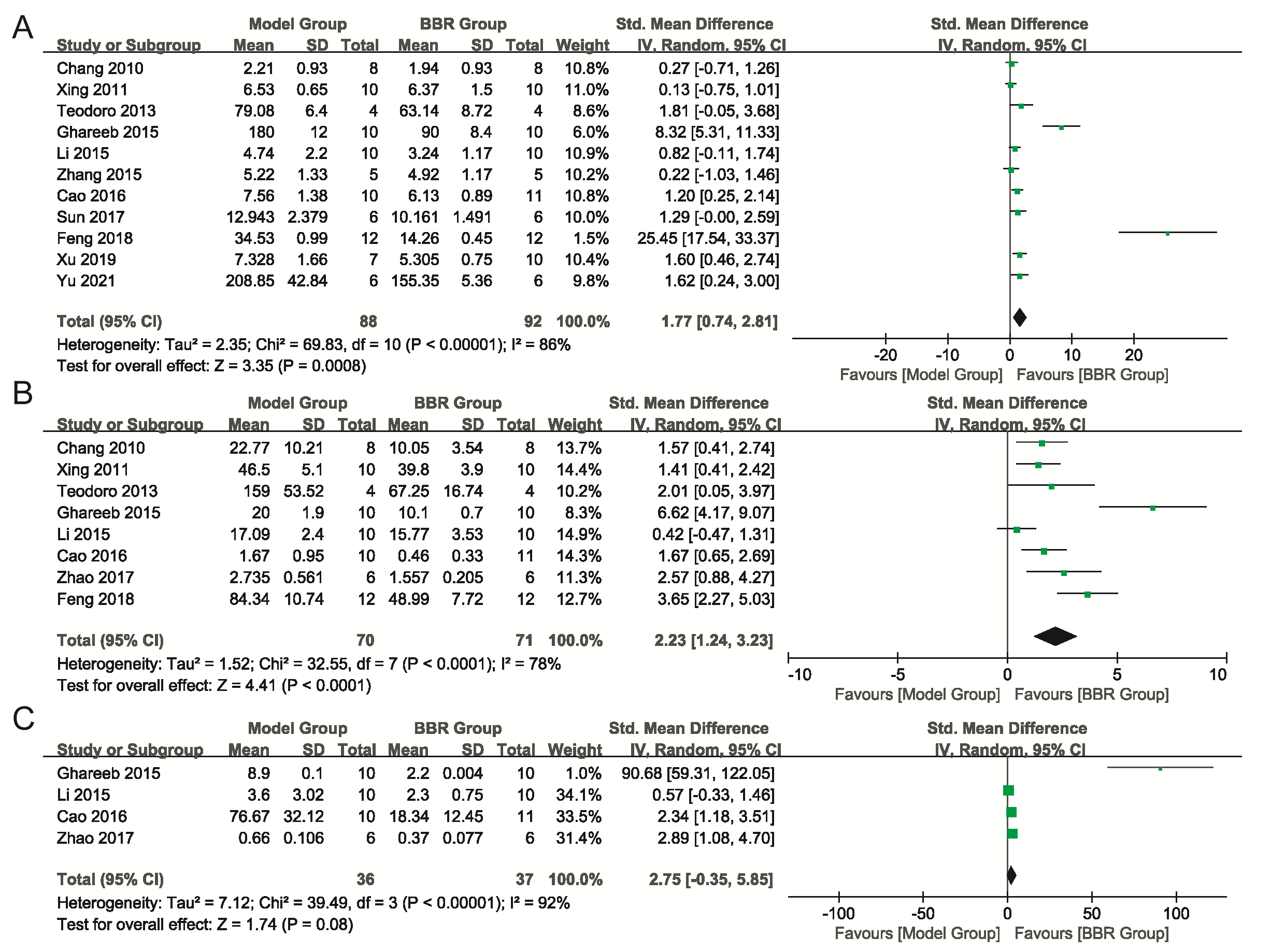
**Supplementary Figure 6. Forest plot of comparison.** (A) FBG; (B) FINS; (C) HOMA-IR; *I^2^* and *P* were taken as the heterogeneity test of the criterion. FBG: fasting blood glucose; FINS: fasting insulin; HOMA-IR: homeostasis model assessment-insulin resistance.


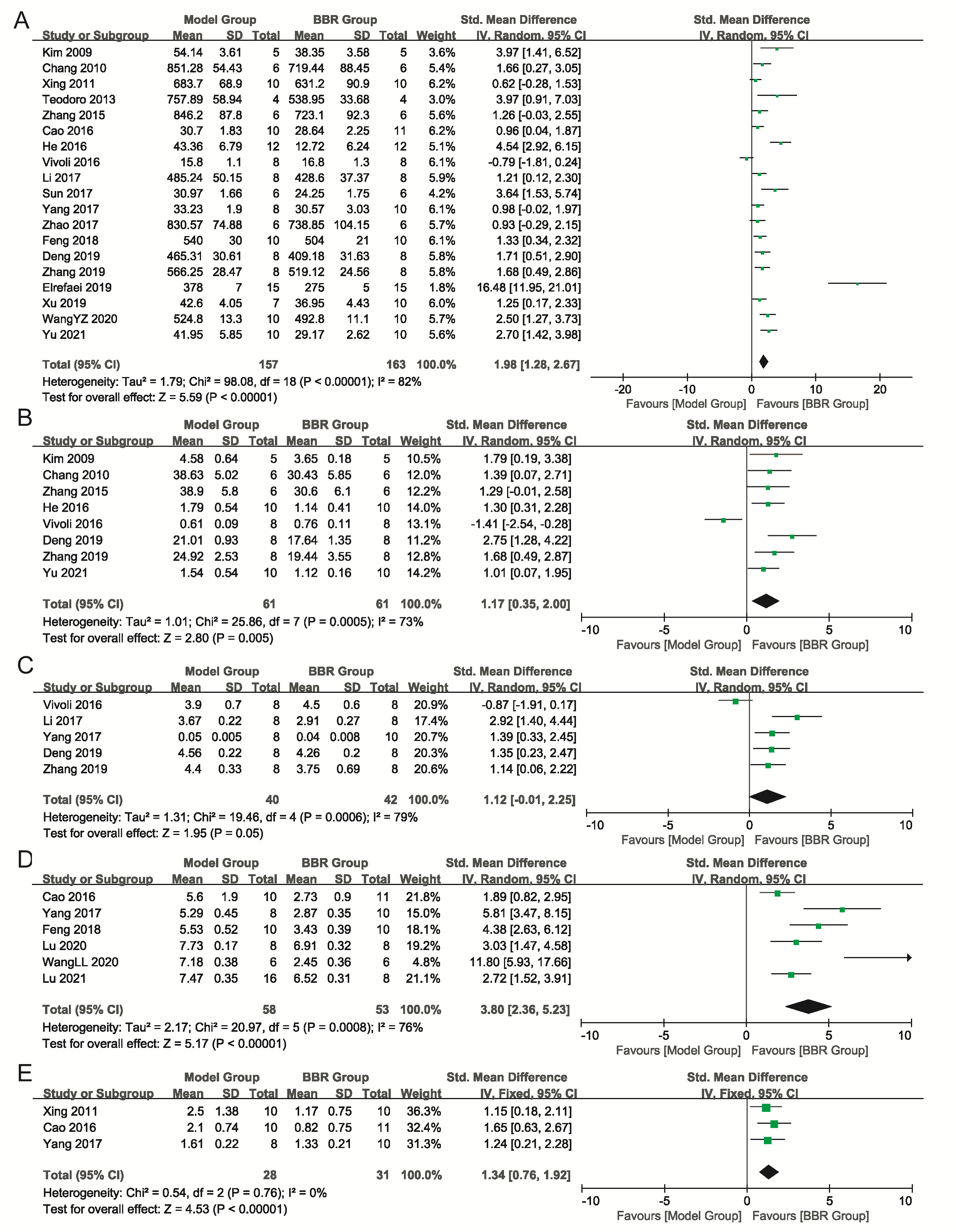


**Supplementary Figure 7.** **Forest plot of comparison.** (A) Body weight; (B) Liver weight; (C) Liver index; (D) NAS; (E) Steatosis score; *I^2^* and *P* were taken as the heterogeneity test of the criterion. NAS: NAFLD activity score.


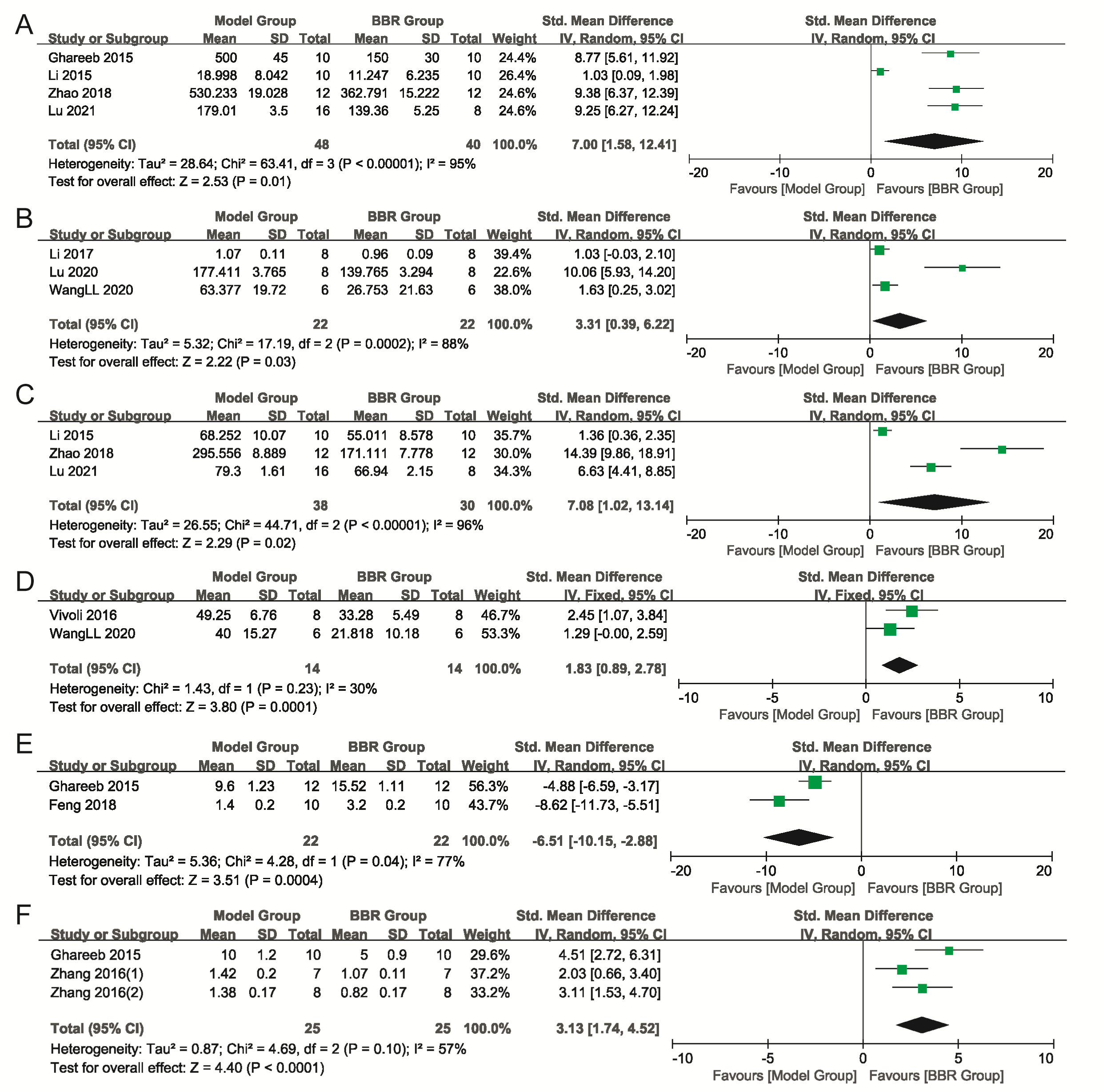


**Supplementary Figure 8.** **Forest plot of comparison.** (A) TNF-α; (B) Liver TNF-α; (C) IL-6; (D) IL-1β; (E) GSH; (F) TBARS; *I^2^* and *P* were taken as the heterogeneity test of the criterion. TNF-α: tumor necrosis factor-α; IL-6: interleukin-6; IL-1β: interleukin-1β; GSH: glutathione; TBARS: thiobarbituric acid reactive substances.


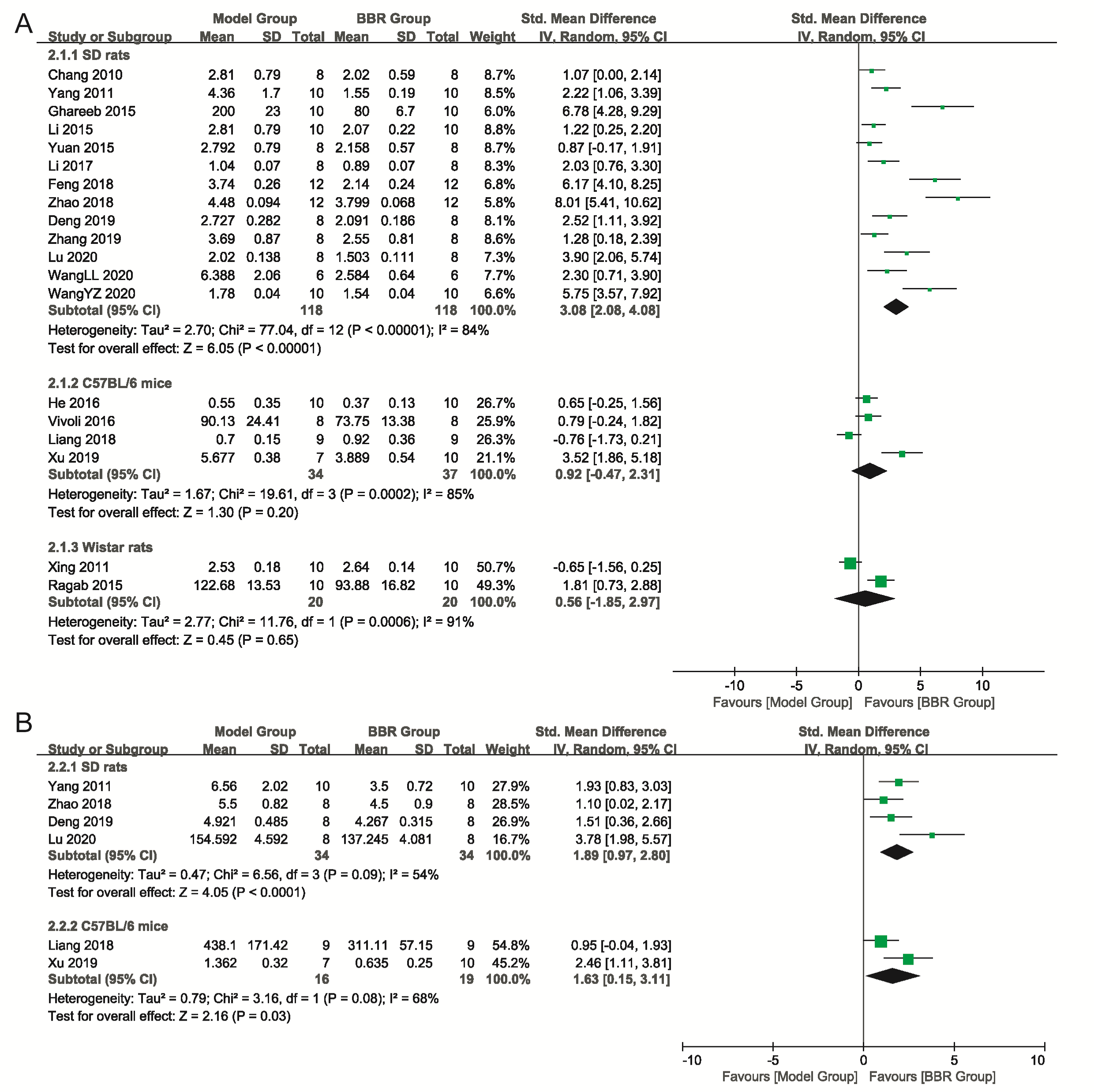


**Supplementary Figure 9.** **Forest plot of comparison for subgroup of different animal breeds.** (A) Blood TC; (B) Liver tissue TC; *I^2^* and *P* were taken as the heterogeneity test of the criterion. TC: total cholesterol.


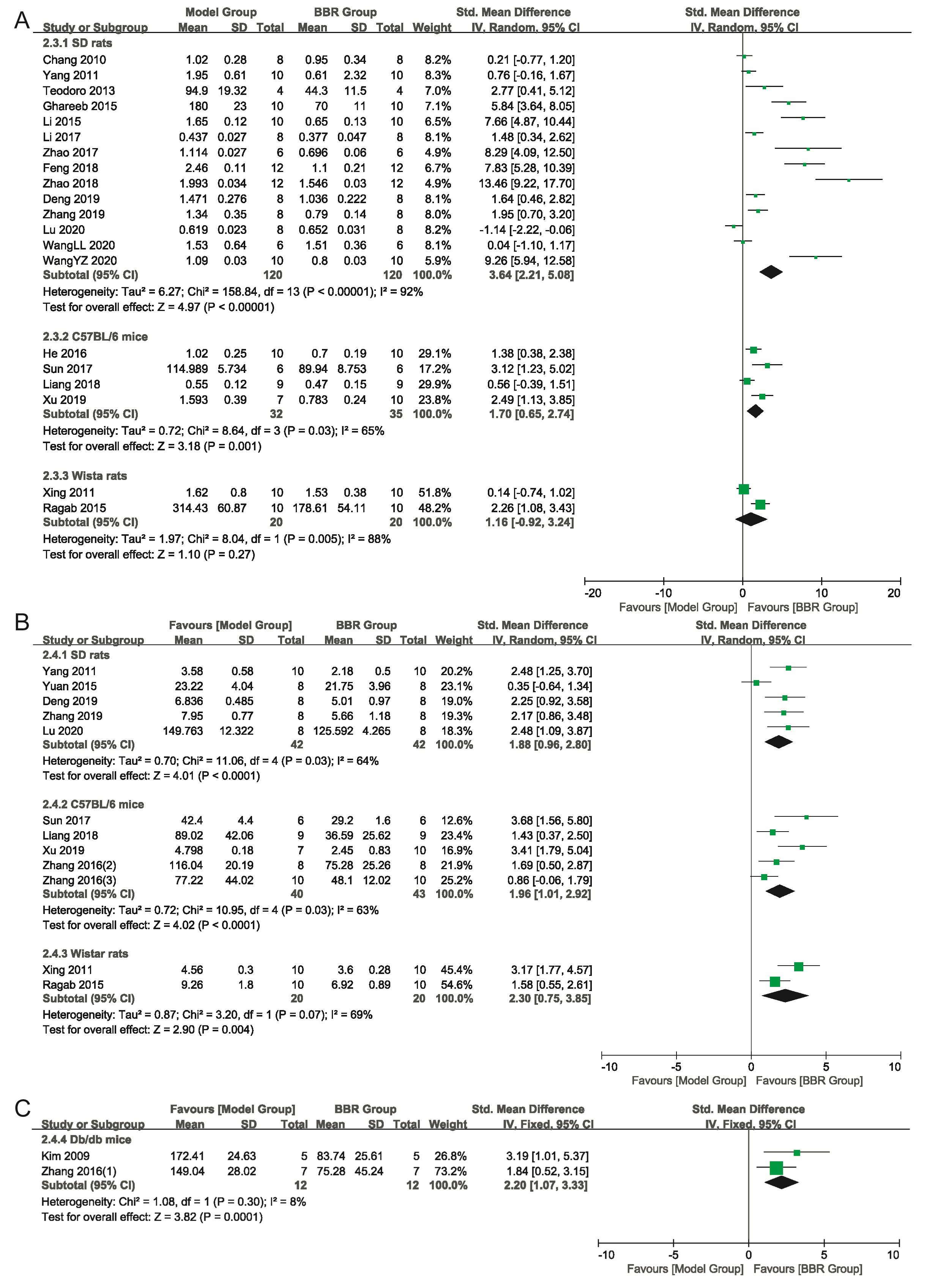


**Supplementary Figure 10.** **Forest plot of comparison for subgroup of different animal breeds.** (A) Blood TG; (B) Liver tissue TG; *I^2^* and *P* were taken as the heterogeneity test of the criterion. TG: triglycerides.


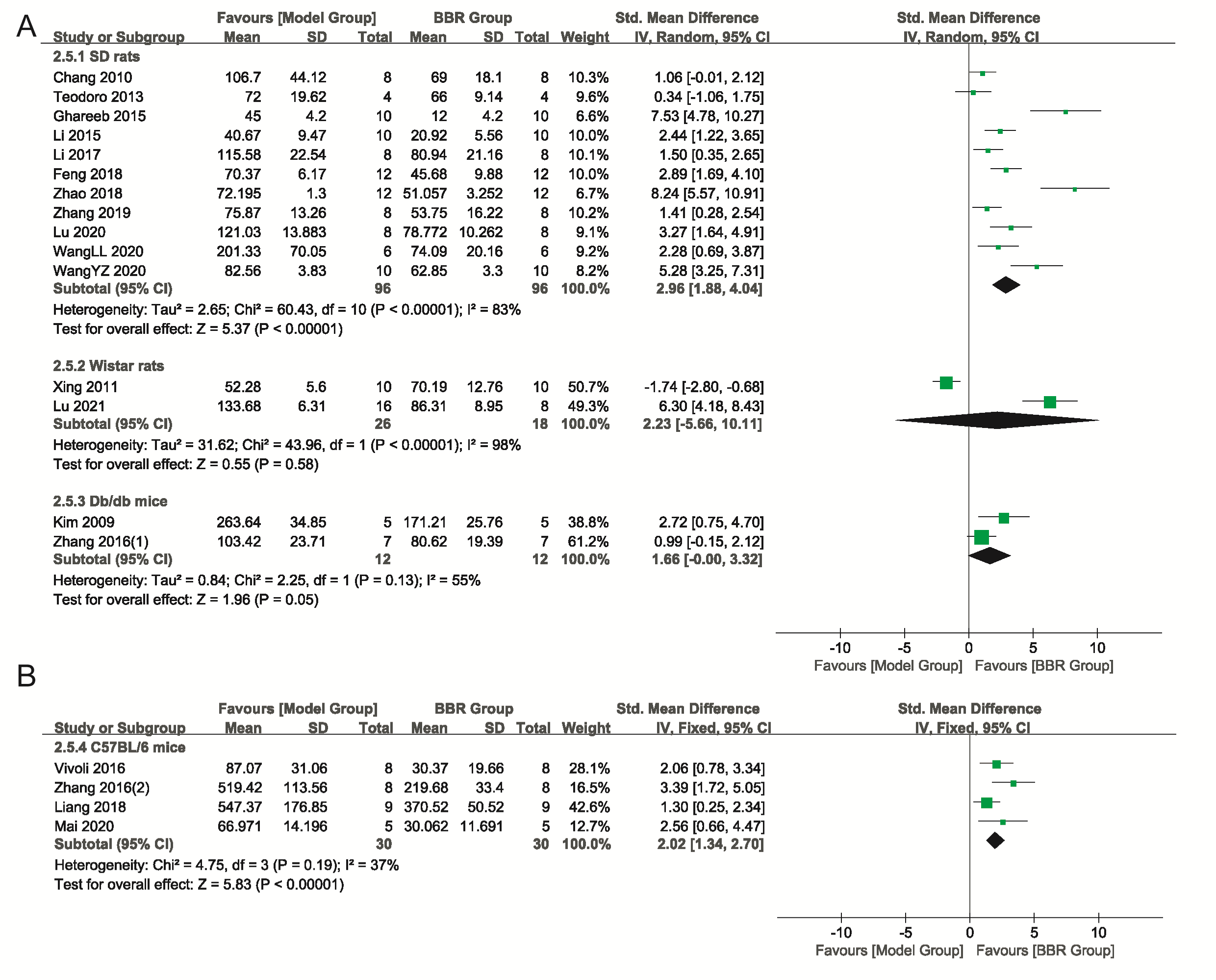


**Supplementary Figure 11.** **Forest plot of comparison for subgroup of different animal breeds.** (A-B) ALT; *I^2^* and *P* were taken as the heterogeneity test of the criterion. ALT: alanine aminotransferase.


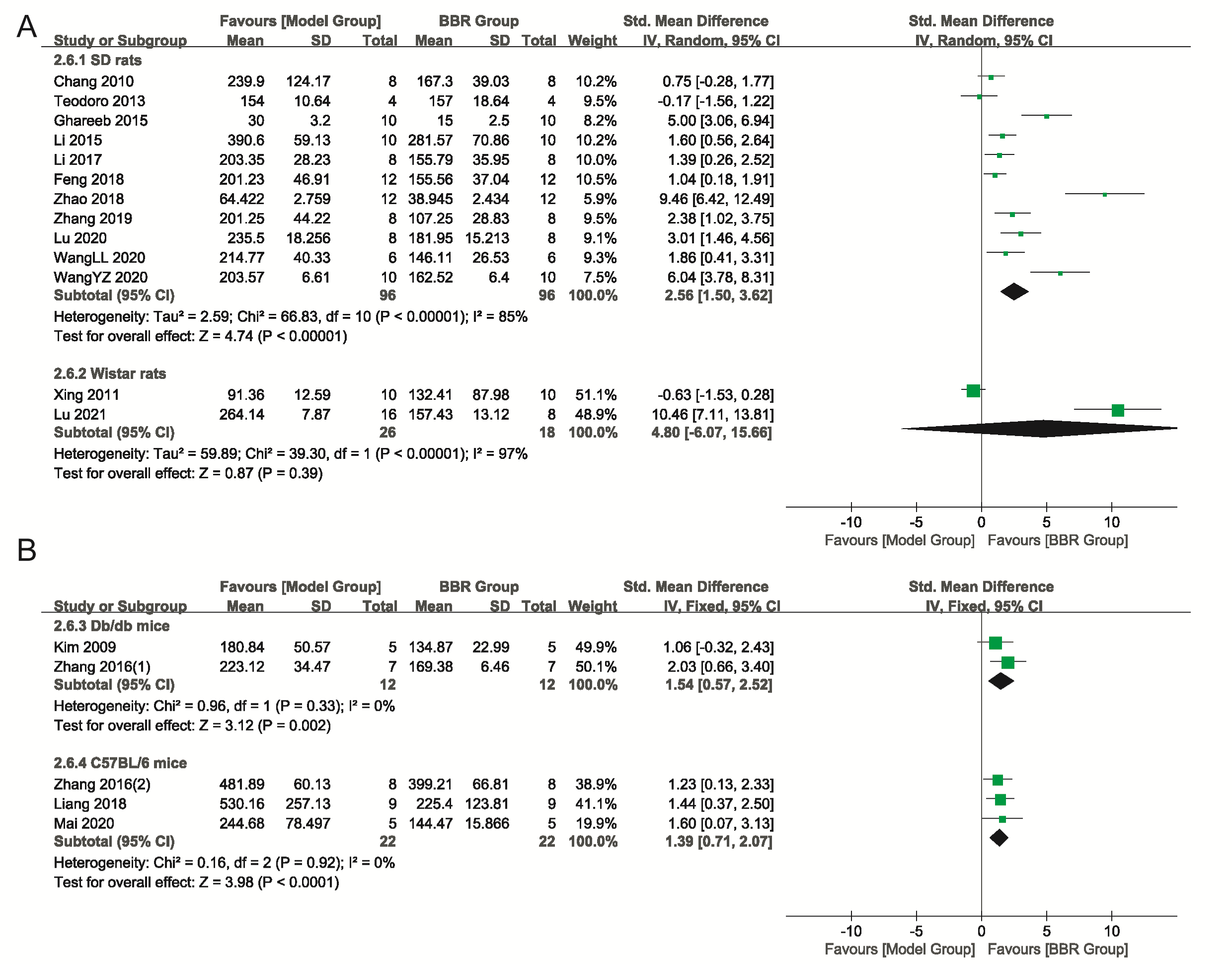


**Supplementary Figure 12.** **Forest plot of comparison for subgroup of different animal breeds.** (A-B) AST; *I^2^* and *P* were taken as the heterogeneity test of the criterion. AST: aspartate amino-transferase.


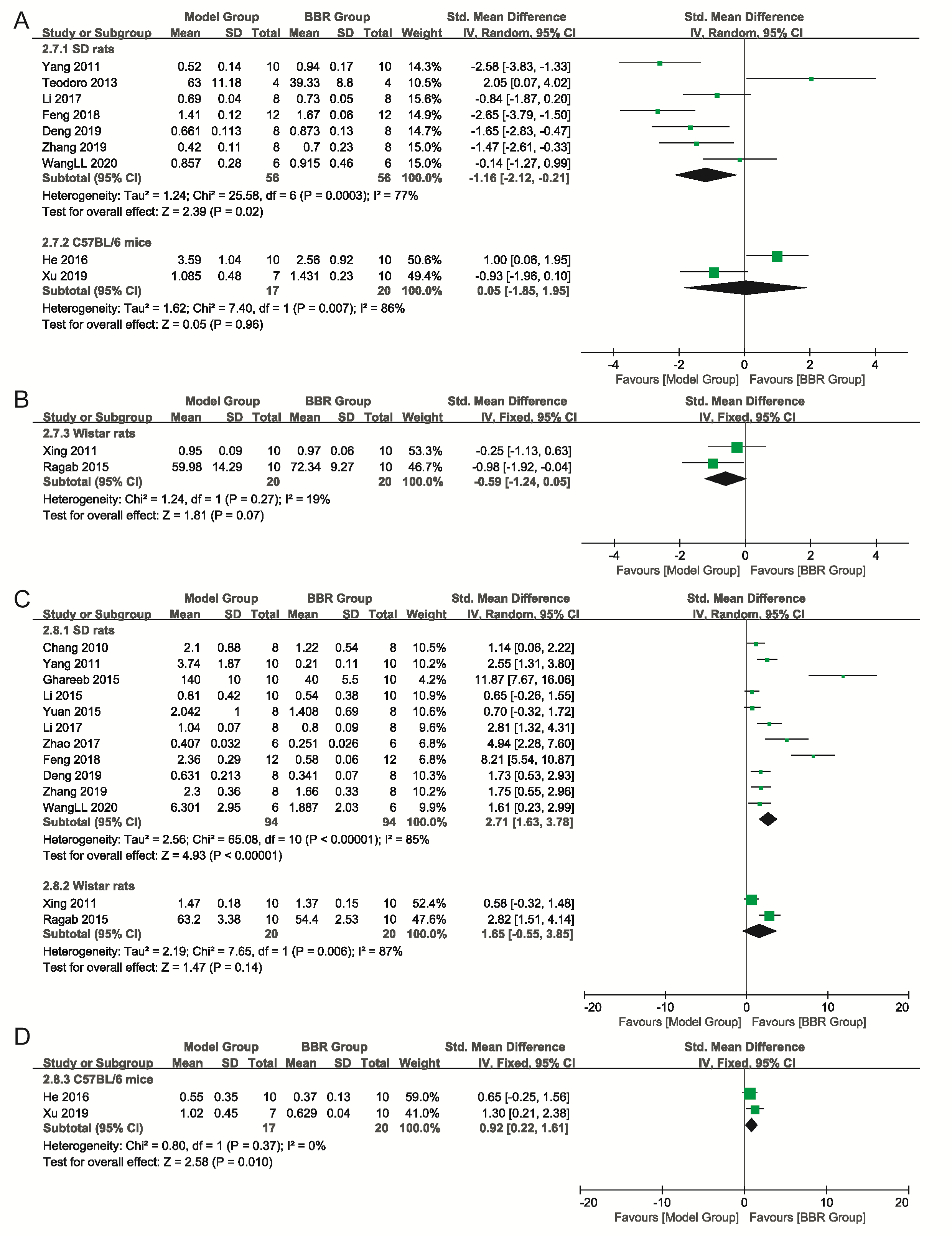


**Supplementary Figure 13.** **Forest plot of comparison for subgroup of different animal breeds.** (A-B) HDL-C; (C-D) LDL-C. *I^2^* and *P* were taken as the heterogeneity test of the criterion. HDL-C: high-density lipoprotein cholesterol; LDL-C: Low-density lipoprotein cholesterol.


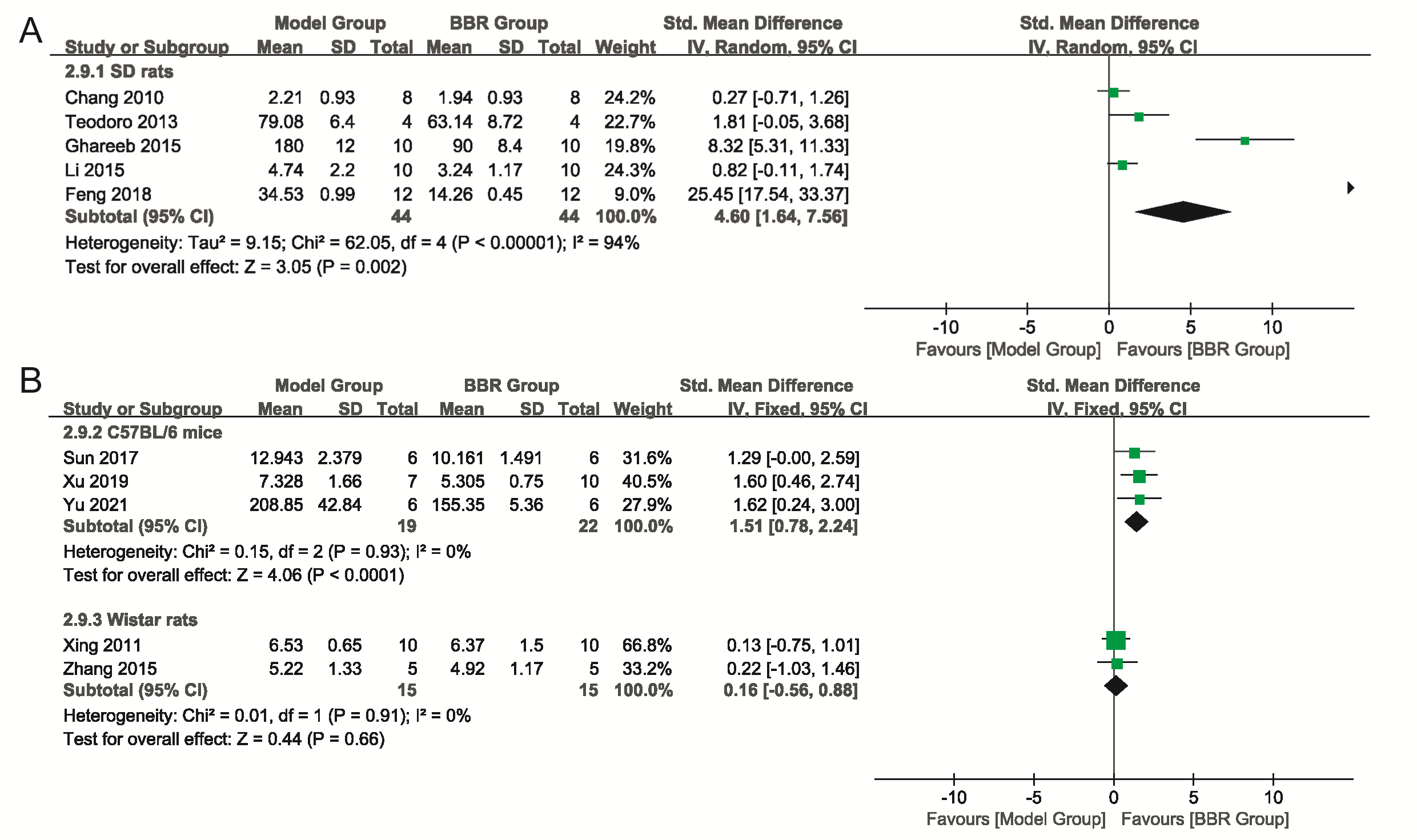
**Supplementary Figure 14.** **Forest plot of comparison for subgroup of different animal breeds.** (A-B) FBG; *I^2^* and *P* were taken as the heterogeneity test of the criterion. FBG: fasting blood glucose.


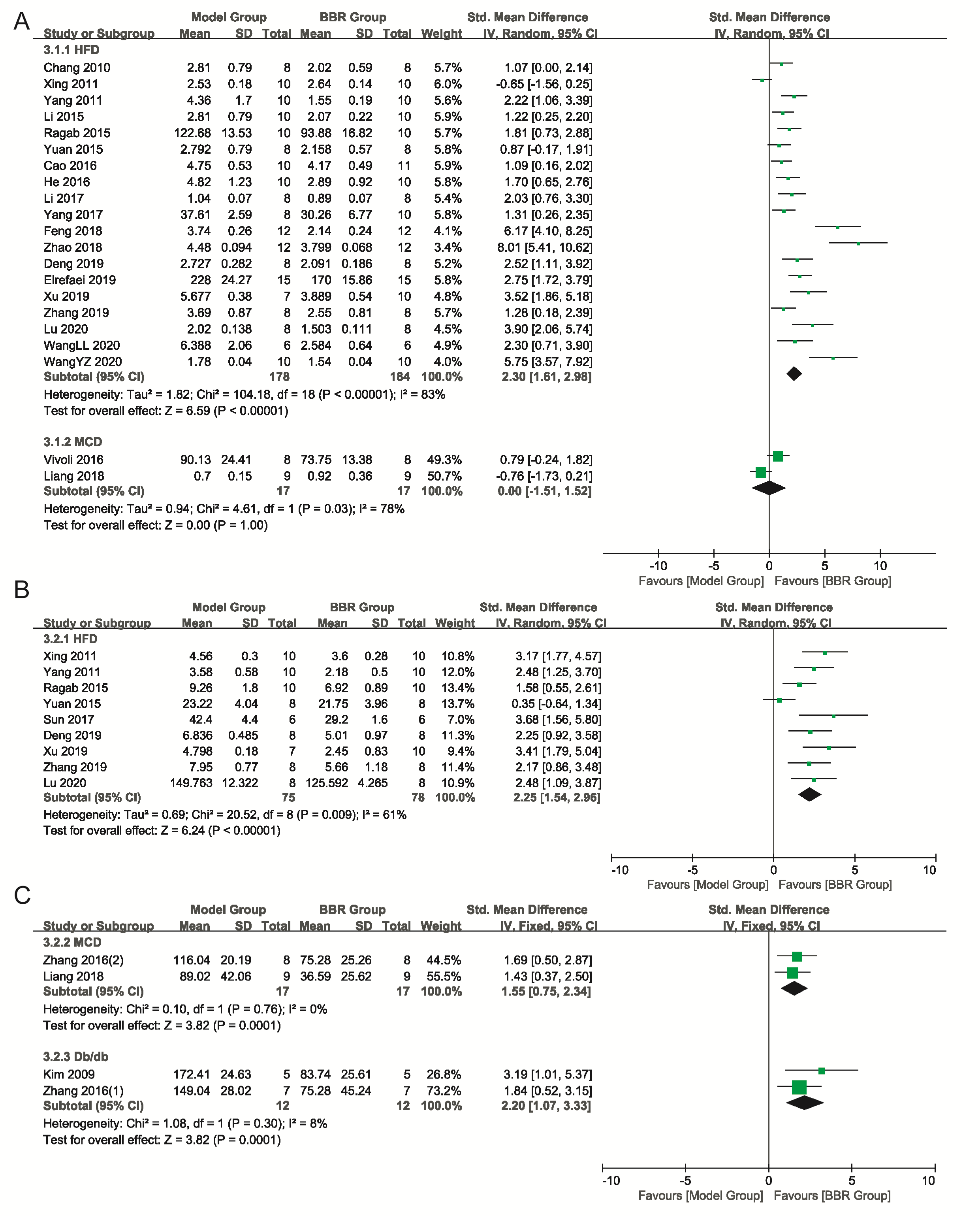


**Supplementary Figure 15.** **Forest plot of comparison for subgroup of different animal models.** (A) Blood TC; (B-C) Liver tissue TG; *I^2^* and *P* were taken as the heterogeneity test of the criterion. TC: total cholesterol; TG: triglycerides.


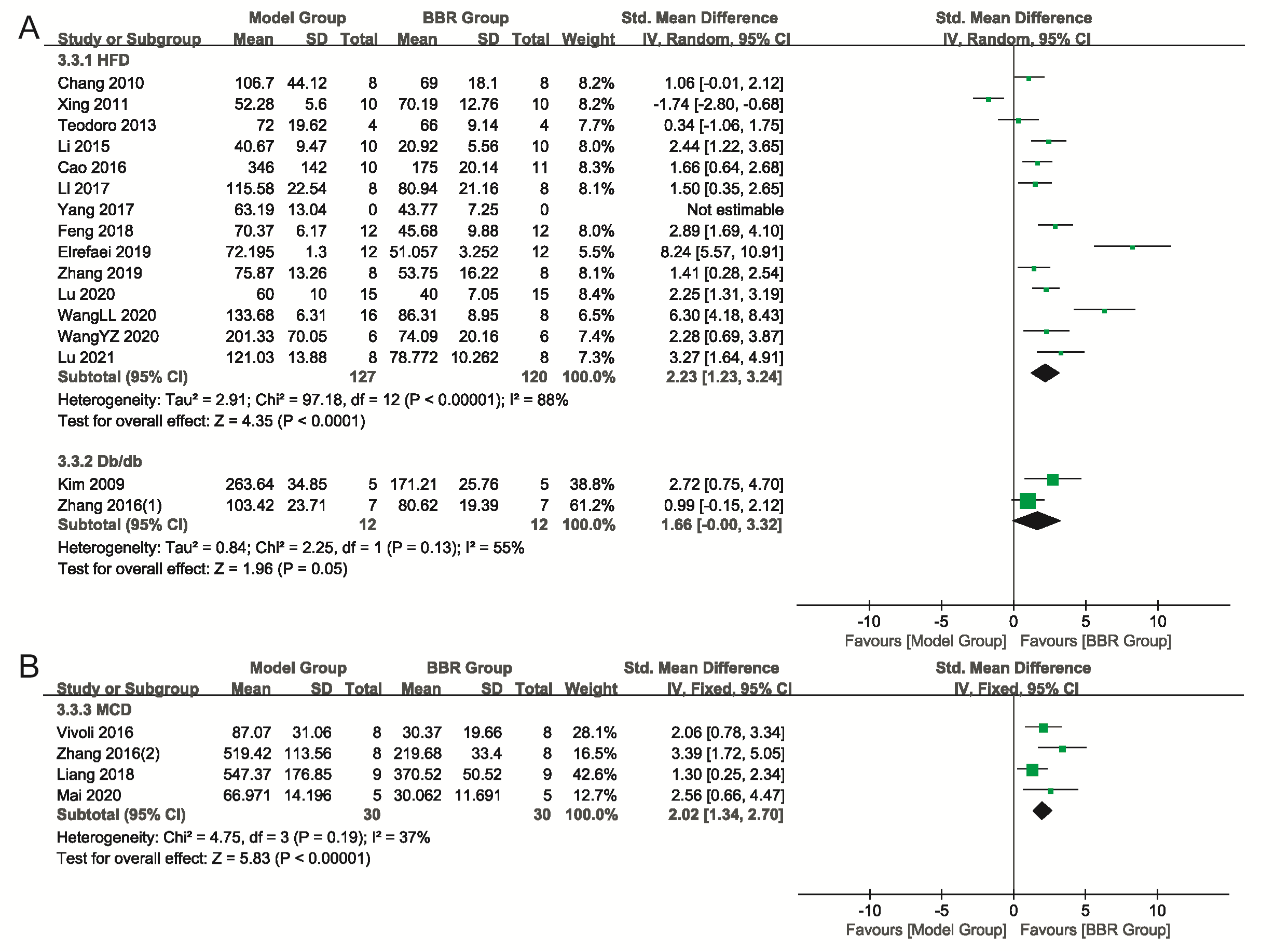


**Supplementary Figure 16. Forest plot of comparison for subgroup of different animal models.** (A-B) ALT; *I^2^* and *P* were taken as the heterogeneity test of the criterion. ALT: alanine aminotransferase.


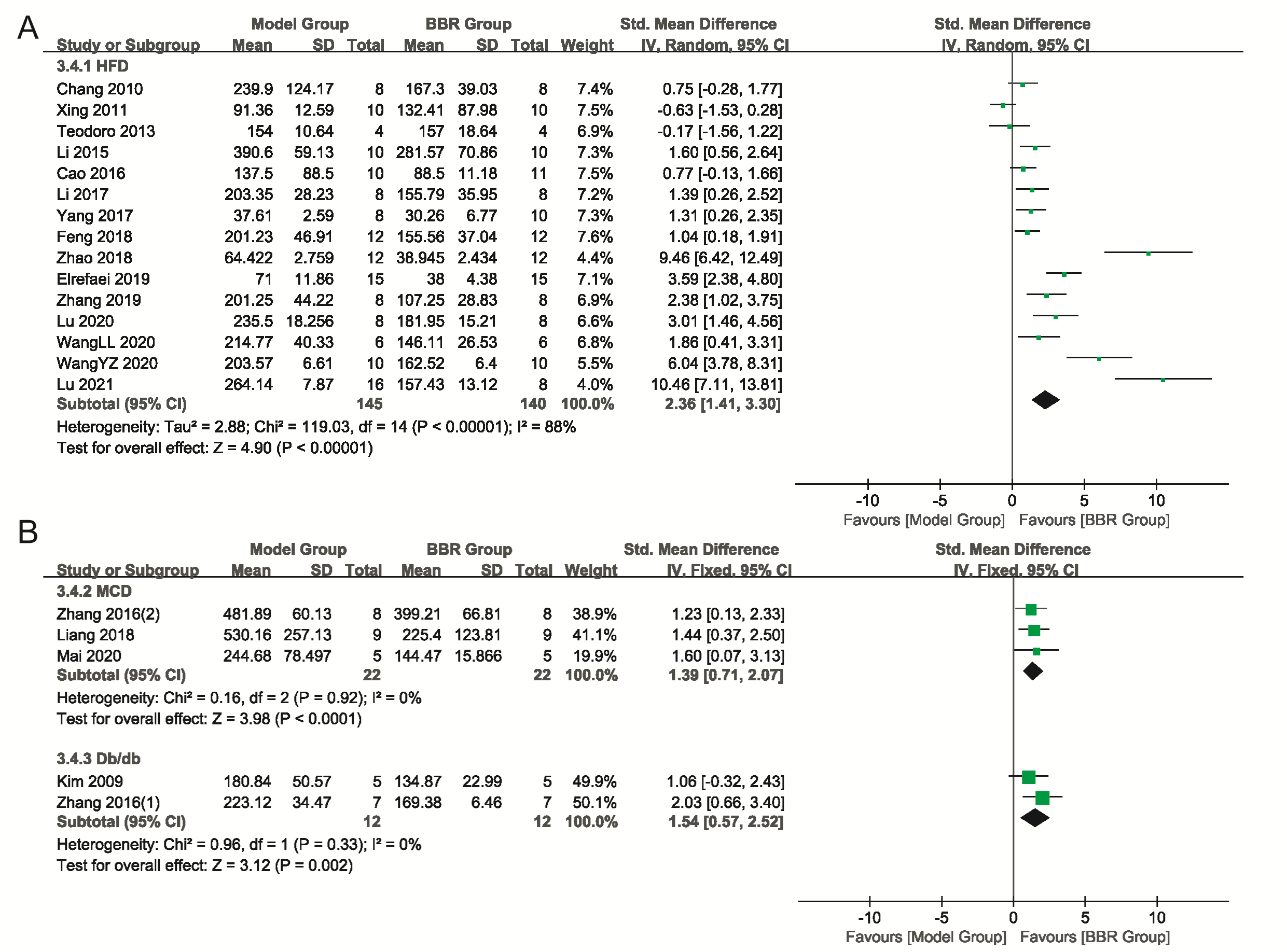


**Supplementary Figure 17.** **Forest plot of comparison for subgroup of different animal models.** (A-B) AST; *I^2^* and *P* were taken as the heterogeneity test of the criterion. AST: aspartate amino-transferase.


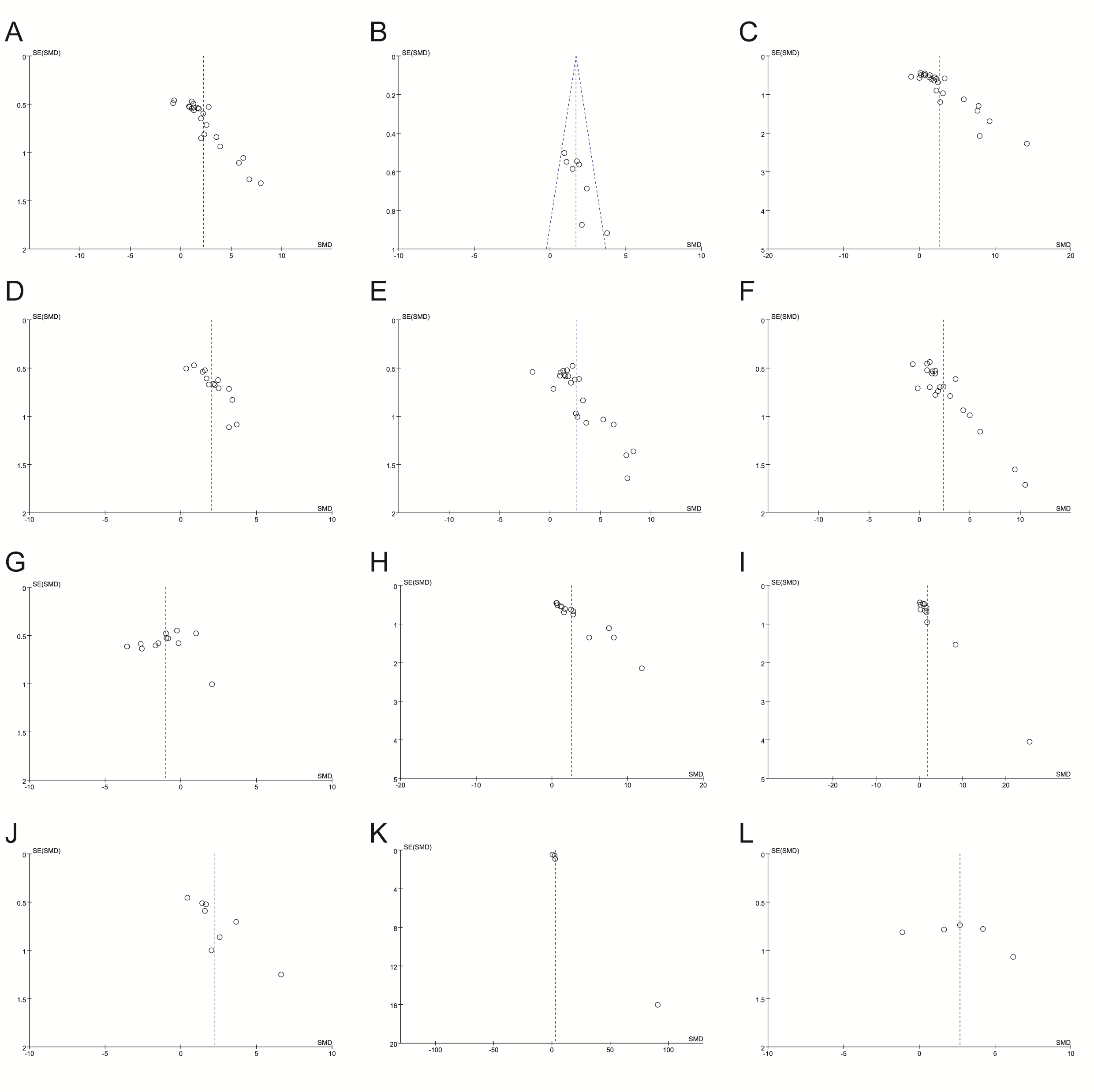
**Supplementary Figure 18. Funnel plot for the publication bias.** (A) blood TC; (B) liver tissue TC; (C) blood TG; (D) liver tissue TG; (E) ALT; (F) AST; (G) HDL-C; (H) LDL-C; (I) FBG; (J) FINS; (K) HOMA-IR; (L) FFA;

**Supplementary Table 1. The characteristics of the included studies**

| Author (years) | Special/strain | Model group | BBR group | N (M/T) | Outcome measures | Secondary outcomes |
| --- | --- | --- | --- | --- | --- | --- |
| Kim et al., 2009 | mice/Db/db | Db/db | 5mg/kg i.p., 3w | 10(5/5) | blood TG, TC, FFA, ALT, AST; tissue TG, TC | body weight, liver weight |
| Chang et al., 2010 | rat/SD | HFD, 24w | 200mg/kg, 16w | 16(8/8) | blood TC, LDL-C, TG, ALT, AST, FBG, FIN | body weight, liver weight |
| Xing et al., 2011 | rat/Wistar | HFD, 8w | 187.5mg/kg, 4w | 20(10/10) | blood ALT, AST, TG, TC, HDL-C, LDL-C, FBG FIN; tissue TG | body weight, steatosis score |
| Yang et al., 2011 | rat/SD | HFD, 10w | 162/324mg/kg, 12w | 20(10/10) | blood TG, TC, HDL-C, LDL-C; tissue TG, TC |  |
| Teodoro et al., 2013 | rat/SD | HFD, 12w | 100mg/kg, 4w | 8(4/4) | blood HDL-C, LDL-C, FFA, TG, ALT, AST, FBG, FIN, | body weight |
| Ghareeb et al., 2015 | rat/SD | 50μl/kg CCl_4_ i.p, 3 times/w, 3w | 50mg/kg, 3w | 20(10/10) | blood AST, ALT, TG, TC, LDL-C, FBG, FIN, HOMA-IR | TNF-α, GSH, TBARS |
| Li et al., 2015 | rat/SD | HFD | 200mg/kg, 8w | 20(10/10) | blood ALT, AST, TG, TC, LDL, FBG, FIN, HOMA-IR | TNF-α, IL-6 |
| Ragab et al., 2015 | rat/Wistar | HFD, 6w | 50mg/kg, 6w | 20(10/10) | blood TC, TG, HDL-C, LDL-C; tissue TC, TG |  |
| Yuan et al., 2015 | rat/SD | HFD, 24w | 200mg/kg, 16w | 16(8/8) | blood TC, LDL-C; tissue TG |  |
| Zhang et al., 2015a | rat/Wistar | HFD, 24w | 200mg/kg, 16w | 10(5/5) | blood FSG | body weight, liver weight |
| Cao et al., 2016 | mice/BALB/c | HFD, 13w | 200mg/kg, 8w | 21(10/11) | blood TC, TG, ALT, AST, FBG, FIN, HOMA-IR | body weight, NAS, steatosis score |
| He et al., 2016 | mice/C57BL/6 | HFD, 12w | 50mg/kg, 4w | 20(10/10) | blood TC, TG, HDL-C, LDL-C | body weight, liver weight |
| Vivoli et al., 2016 | mice/C57BL/6 | MCD, 28d | 5mg/kg i.p., 28d | 16(8/8) | blood ALT, TC | body weight, liver weight, liver index, tissue IL-1β |
| Zhang et al., 2016 | mice/Db/db | Db/db | 200mg/kg, 5w | 14(7/7) | tissue TG, ALT, AST | TBARS |
|  | mice/C57BL/6 | MCD, 2w | 200mg/kg, 4w | 16(8/8) | tissue TG, ALT, AST | TBARS |
|  | mice/C57BL/6 | TM (1 mg/kg) i.p., 24h | 200mg/kg, 3d | 20(10/10) | tissue TG |  |
| Li et al., 2017 | rat/SD | HFD, 8w | 150mg/kg, 4w | 16(8/8) | blood TG, TC, HDL-C, LDL, ALT, AST | body weight, liver index, tissue TNF-α |
| Sun et al., 2017 | mice/C57BL/6 | HFD | 150mg/kg, 8w | 12(6/6) | blood FSG, TG; tissue TG | body weight |
| Yang et al., 2017 | mice/C57BL/6 J ApoE^-/-^ | HFD | 200mg/kg | 18(8/10) | blood ALT, AST, TC, TG | body weight, liver index, NAS, steatosis score |
| Zhao et al., 2017 | rat/SD | HFD,16w | 150mg/kg, 16w | 12(6/6) | blood TG, LDL-C, FINS, HOMA-IR | body weight |
| Feng et al., 2018 | rat/SD | HFD, 12w | 100mg/kg, 8w | 24(12/12) | blood TC, TG, HDL-C, LDL-C, FFA, AST, ALT, FSG, FIN, | body weight, NAS, GSH |
| Liang et al., 2018 | mice/C57BL/6 | MCD,6w | 200mg/kg, 4w | 18(9/9) | blood ALT, AST, TC, TG; tissue TG, TC |  |
| Zhao et al., 2018 | rat/SD | HFD, 4w | 250mg/kg, 4/8w | 24(12/12) | blood ALT, AST, TC, TG, | TNF-α, IL-6 |
| Deng et al., 2019 | rat/SD | HFD, 8w | 100mg/kg, 8w | 16(8/8) | blood TC, TG, HDL-C, LDL-C; Tissue TC, TG | body weight, liver weight, liver index, GSH |
| Elrefaei et al., 2019 | rat/albino | HFD, 6w | 200mg/kg, 6w | 30(15/15) | blood ALT, AST, TG, TC, HDL-C, LDL-C | body weight |
| Xu et al., 2019 | mice/C57BL/6 | HFD,8w | 300mg/kg, 4w | 17(7/10) | blood TG, TC, LDL-C, HDL-C, FBG; tissue TC, TG | body weight |
| Zhang et al., 2019 | rat/SD | HFD | 100mg/kg, 8/16w | 16(8/8) | blood TC, TG, HDL-C, LDL-C, ALT, AST; tissue TC, TG | body weight, liver weight, liver index |
| Lu et al., 2020 | rat/SD | HFD, 4w | 300mg/kg, 8w | 16(8/8) | blood ALT, AST, TC, TG, FFA; tissue TG, TC | NAS, tissue TNF-α |
| Mai et al., 2020 | mice/C57BL/6 | MCD, 5w | 100mg/kg, 2w | 10(5/5) | blood ALT, AST, |  |
| WangLL et al., 2020 | rat/SD | HFD, 12w | 200mg/kg, 12w | 24(12/12) | blood ALT, AST, TG, TC, HDL-C, LDL-C; | NAS, tissue TNF-α, IL-1β |
| WangYZ et al., 2020 | rat/SD | HFD, 12w | 150mg/kg, 4W | 22(11/11) | blood ALT, AST, TC, TG, | body weight, IL-1βTNF-α |
| Lu et al., 2021 | rat/Wistar | HFD, 4w | 300mg/kg, 8w | 24(16/8) | blood ALT, AST, FFA | NAS, TNF-α, IL-6 |
| Yu et al., 2021 | mice/C57BL/6J | HFD, 20w | 1.4g/kg blended into the HFD, 20w | 20(10/10) | blood FSG | body weight, liver weight |

**Abbreviations:** BBR: berberine; N(M/B): number (model/BBR group); Db/db: Obese and diabetic C57BLKS/J-*Lepr*^db^/*Lepr*^db^ mice; TM: tunicamycin; SD: Sprague–Dawley; HFD: high-fat diet; MCD: methionine and choline-deficient diet;
